# The northernmost haulout site of South American sea lions and fur seals in the western South Atlantic

**DOI:** 10.1038/s41598-020-76755-2

**Published:** 2020-11-17

**Authors:** Natália Procksch, M. Florencia Grandi, Paulo Henrique Ott, Karina Groch, Paulo A. C. Flores, Marcelo Zagonel, Enrique A. Crespo, Rodrigo Machado, Guido Pavez, Murilo Guimarães, Maurício Veronez, Larissa Rosa de Oliveira

**Affiliations:** 1 Laboratório de Ecologia de Mamíferos (LEM), Universidade Do Vale Do Rio Dos Sinos (UNISINOS), Av. Unisinos 950, Cristo Rei, São Leopoldo, RS 93022-750 Brazil; 2 Laboratório de Mamíferos Marinos, Centro Para El Estudio de Sistemas Marinos, CONICET, Bvd. Brown 2915, 9120 Puerto Madryn, Chubut Argentina; 3 Laboratório de Biodiversidade E Conservação (LABeC), Universidade Estadual Do Rio Grande Do Sul (UERGS), Rua Machado de Assis, 1456, Osório, RS 95520-000 Brazil; 4 Grupo de Estudos de Mamíferos Aquáticos Do Rio Grande Do Sul (GEMARS), Rua Bento Gonçalves, 165, sala 1002, Torres, RS 95560-000 Brazil; 5 Projeto Baleia Franca, Instituto Australis de Pesquisa E Monitoramento Ambiental, Av. Atlântica, s/n- Itapiruba Norte, Imbituba, SC 88780-000 Brazil; 6 Centro Mamíferos Aquáticos, Currently at Área de Proteção Ambiental (Environmental Protection Area) Anhatomirim-SC, ICMBio-MMA, Rod. SC 402, km 1, Jurerê, Florianópolis, SC 88053-700 Brazil; 7 Laboratório de Ecologia Espacial, Universidade Do Vale Do Rio Dos Sinos (UNISINOS), Av. Unisinos 950, Cristo Rei, São Leopoldo, RS 93022-750 Brazil; 8 Advanced Visualization and Geoinformatics Laboratory (VizLab), Universidade Do Vale Do Rio Dos Sinos (UNISINOS), Av. Unisinos 950, Cristo Rei, São Leopoldo, RS 93022-750 Brazil; 9 Centro de Investigación Y Gestión en Recursos Naturales (CIGREN), Instituto de Biología, Facultad de Ciencias, Universidad de Valparaíso, Gran Bretaña 1111, Playa Ancha, Valparaíso, Chile; 10 Departamento de Zoologia, Universidade Federal Do Rio Grande Do Sul, Avenida Bento Gonçalves, 9500, Agronomia, Porto Alegre, RS 91509-900 Brazil

**Keywords:** Conservation biology, Marine biology, Ecology, Zoology

## Abstract

We present estimates of the seasonal and spatial occupation by pinnipeds of the Wildlife Refuge of Ilha dos Lobos (WRIL), based on aerial photographic censuses. Twenty aerial photographic censuses were analysed between July 2010 and November 2018. To assess monthly differences in the numbers of pinnipeds in the WRIL we used a Generalized Linear Mixed Model. Spatial analysis was carried out using Kernel density analysis of the pinnipeds on a grid plotted along the WRIL. Subadult male South American sea lions (*Otaria flavescens*) were the most abundant pinniped in the WRIL. Potential females of this species were also recorded during half of the census. The maximum number of pinnipeds observed in the WRIL was 304 in September 2018, including an unexpected individual southern elephant seal (*Mirounga leonina*), and a high number of South American fur seal yearlings (*Arctocephalus australis*). However, there was no statistically significant difference in counts between months. In all months analysed, pinnipeds were most often found concentrated in the northern portion of the island, with the highest abundances reported in September. This study confirms the importance of the WRIL as a haulout site for pinnipeds in Brazil, recommends that land research and recreational activities occur in months when no pinnipeds are present, and encourages a regulated marine mammal-based tourism during winter and spring months.

## Introduction

Every year hundreds of pinnipeds of various species are found along the southern Brazilian coast, most of which are South American fur seals (*Arctocephalus australis*) and South American sea lions (*Otaria flavescens*)^1–4^. Antarctic and Subantarctic pinnipeds such as Antarctic fur seals (*Arctocephalus gazella*), Subantarctic fur seals (*Arctocephalus tropicalis*), leopard seals (*Hydrurga leptonyx*), and southern elephant seals (*Mirounga leonina*) are also reported in the region, but they are considered occasional or rare visitors^4–6^. Recently, the first record of a Weddell seal (*Leptonychotes weddellii*) in Brazilian waters was reported for Trindade Island^7^, and the presence of fur seal (*Arctocephalus* sp.) in the equatorial Atlantic Ocean was recorded for the São Pedro and São Paulo Archipelago, which is about 1010 km away from the Brazilian mainland, just north of the Equator^8^.

**Figure 1:**
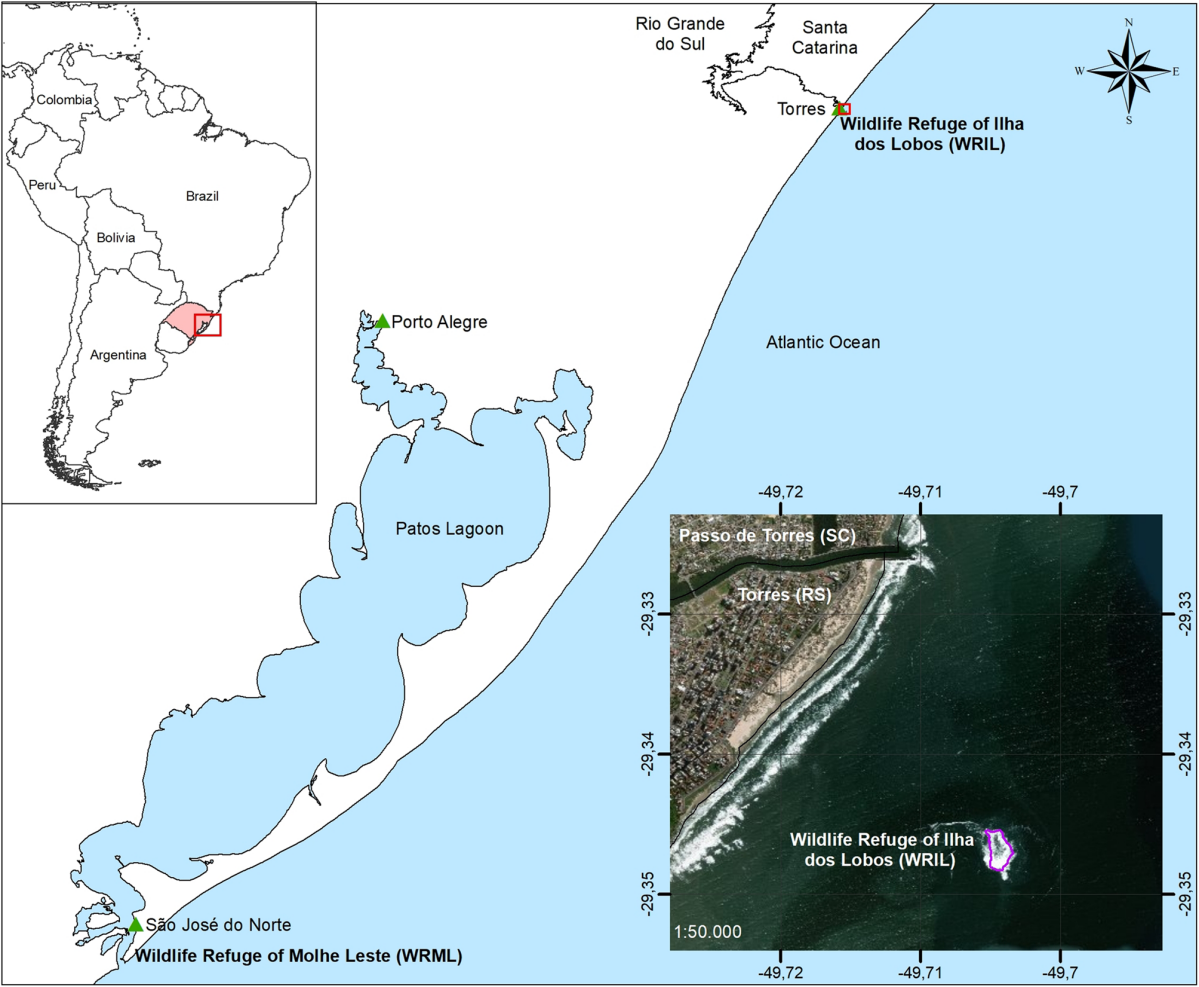
Map of the study area, showing the Wildlife Refuge of Ilha dos Lobos on the southern Brazilian coast, with the emerged area of the island marked in purple (generated in ArcMap 10.6.1). The protection limits of this marine protected area are not shown in the figure.

Brazil has no breeding colonies for any species of pinniped and only two haulout sites that are within marine protected areas (MPAs): the Wildlife Refuge of Molhe Leste of São José de Norte (WRML) (32° 10′ S; 52° 06′ W), and the Wildlife Refuge of Ilha dos Lobos (WRIL) (29° 20′ S; 49° 42′ W), both on the coast of the Rio Grande do Sul, southern Brazil^4,9^ (Fig. 1). WRML is a small area located about 400 km south of WRIL, where sea lions and fur seals rest at the tip of a 4 km long jetty in the mouth of the Patos Lagoon estuary, close to Rio Grande harbour^2^. The WRIL is the only coastal island in the Rio Grande do Sul state, in southern Brazil.

The South American pinnipeds found in Brazil (*A. australis* and *O. flavescens*) probably arrive from their closest breeding sites in Uruguay and Argentina^1,2^, mainly during the austral autumn and spring months^2–4,10^. The two species have similar reproductive periods during the austral summer, with South American sea lions breeding from December to early February^11^, and South American fur seals breeding from late November to mid-January^12,13^. Males of both species do not provide paternal care and once the breeding period ends, they move away from their rookeries to return in the next breeding season. Meanwhile, females divide their time between foraging trips and fasting periods while nursing, therefore presenting more limited movements^2,13,14^. The arrival and residence of these species in the WRIL corresponds to the dispersal period after the breeding season, which is the least understood period of the life-cycle of these species^15,16^. Recent tagging^17^ (Andre Barreto, UNIVALI, pers. comm.) and genetic studies^18,19^ have confirmed that South American fur seals and South American sea lions from Argentine and Uruguayan colonies disperse northwards to Brazil.

The International Union for the Conservation of Nature (IUCN) considers the South American sea lion as a species of Least Concern^20^ at a global level, and the different populations on the Atlantic coast of South America have contrasting trends. The total abundance of this species, including on the Pacific and Atlantic coasts of South America, was estimated to be 445,000 individuals in 2012^20^. While most of the populations are increasing in abundance, the Uruguayan population has been decreasing at roughly 2% per year^21,22^. The Uruguayan population is concentrated on the Isla de Lobos (35° 01′ S; 54° 52′ W) and at Cabo Polonio (34° 24′ S; 53° 47′ W), and is estimated to number between 12,000 and 13,000 individuals, with an average of 1500 pups born every year^21,22^. Another declining population of South American sea lion is found on the Falklands/Malvinas (51° 45′ S; 59° 00′ W), with 7500 animals found in 2003^23^ and a minimum estimate of 4443 pups born at the in 2014^24^. For the coast of Argentina there is an increasing trend in the population numbers, including breeding and non-breeding colonies, with the total population estimated at about 123,200 individuals^20^.

The most recent published information on the total population of South American fur seals estimated numbers of around 211,000 in 2016 and the current IUCN status of the species is Least Concern^25^. However, taking into account the current pup estimates for the Falkland/Malvinas Islands of 36,500 pups^26^, and considering a rough estimate of four adults per pup^27^, the total population of these islands could be approximately 182,500 individuals. Therefore, the global population is likely to be around 393,500 individuals. The Uruguayan population, which is probably the origin of individuals found in Brazil^18^, was estimated to number 300,000 fur seals in the 1990s^21,28^. However, recently, data based on aerial surveys have suggested that this estimate only be around half of the true populations size in the 1990s^29^. The Argentine population is estimated to be around 25,000 individuals, probably due to the immigration of individuals from Uruguay^30^. The main threats for both the South American sea lion and fur seal are marine habitat degradation and interactions with local fisheries, with the latter especially critical for South American sea lions^20,25^.

The arrival of these species in southern Brazil usually corresponds to the dispersal period after the breeding season, which occurs from December to February, and may be assisted by the cold, northwards flowing, Malvinas Current^1,2,4,16^. This dispersal period towards Brazilian waters is one of the most poorly understood parts of the life-cycle of South American pinnipeds^15,16,31^. Therefore, this study seeks to provide more information on the habitat use and distribution of these species during this period.

Traditionally, pinniped censuses are based on land counts, aerial counts and/or capture-recapture studies at haulout sites (e.g. Ref.^32^), and are either conducted from land (e.g. from fixed point on clifftops), from vessels, with unmanned aerial vehicles (UAV) (e.g. Refs.^33,34^) or using small manned aircraft. Although weather impacts the ability to observe the animals from all platforms, censuses from high clifftops are particularly limited by difficult circumstances such as meteorological conditions or access factors that could result in poor visibility of individuals, counting errors (e.g. caused by animals lying between others or hiding behind rocks)^35^, or even disturbance and dispersion of specimens^36,37^. For these reasons, aerial photographs are preferred for pinniped censuses since they reduce the errors in population abundance estimates^34^. In addition, aerial photographic census methods have been successfully used to estimate population sizes for many terrestrial and marine wildlife species, allowing that individuals can be distinguished and counted directly from images^38,39^.

In southern Brazil however, none of the previous studies on pinniped abundance have used aerial photography. The first pinniped abundance study in the WRIL was conducted more than 20 years ago, between 1994 and 1995^16^. The authors used terrestrial and marine census methods from fixed points and later from vessels. Regular census counts taken from a boat moving slowly around the WRIL were also conducted from 1993 to 2002^10,40,41^.

To effectively manage and conserve a protected area, it is necessary to know not only population abundance, but also the spatial distribution of focal species^42–44^. The importance of WRIL resides in the fact that it is currently considered the northern limit of distribution for both South American fur seals and South American sea lions on the Atlantic coast of South America and provides an important habitat for these species outside the breeding period^4,12,45^. However, according to paleontological studies the distribution range of these species was 230 km further north 5000 years ago, on the southern Brazilian coast^46^. Therefore, the persistence of pinnipeds in the WRIL in the future may be driven by both environmental and anthropogenic factors, which can change rapidly under the current global context. In this changing scenario, the WRIL could be considered as an open-air laboratory for understanding the effects of climate change on the abundance South American fur seals and sea lions, and it must be monitored in order to understand the expansion/contraction of the limits of the distribution of these species as a potential response to climate and environment variations. Moreover, studies combining abundance and spatial distribution analysis over time could be useful for informing future management plans for the WRIL. In particular, such information could be useful for the planning (spatial and temporal) of activities that are compatible with the MPA, including tourism, recreational and educational activities, as well as for delimiting buffer zones since this area is subject to intensive anthropogenic activity^47^.

In this context, this study presents the first spatial data on pinniped occurrence at the WRIL as well as new data on seasonal abundance. We tested the three following hypotheses: (1) that there is a difference in pinniped abundance between months, since this haulout site is occupied mainly in the spring months^2–4,10^, (2) that there is an expected predominance of males, which are not as involved in parental care as females^16,40,48^, and (3) sea lions and fur seals demonstrate spatial preferences, because otariid species prefer protected areas both during breeding^49^ and as haulout sites^16^. Based on these findings related to the seasonal and spatial occupation of the WRIL by pinnipeds, recommendations on when and where potential human activities could occur there are proposed to advise the future management plan for this MPA.

## Methods

### Study area and aerial photographic census

The WRIL consists of a coastal island that is 1800 m from the city of Torres, with an area of 16,966 m^2^ during low tide, and an additional 500 m around the island^50,51^ (Fig. 1).

This study was based on aerial images taken with a helicopter and UAV during 20 flights over the WRIL from 2010 to 2018. The flights were carried out by the *Instituto Australis* which performs aerial censuses of the southern right whale, *Eubalaena australis*, in the region^52^. The flights were conducted between July and November of each year^52^, because this is the period when southern right whales occur in this region^53^. Five of the flights were conducted in July, three in August, six in September and six in November. No flights took place in October or December of any year. The images of the WRIL were obtained for the pinniped census under the Brazilian Biodiversity authorization and information system—SISBIO licenses 69627 and 35606-1. Sampled months corresponded with the historical period of the greatest abundance of South American fur seals and South American sea lions in the WRIL according to the scientific literature (e.g. Refs.^2,10,16,40^).

During flights with a helicopter (for details see^52^) aerial photographs with a zoom range of 100–400 mm were taken by a photographer with a Canon EOS 7D digital camera. The flights were carried out at approximately 100–250 m of altitude in order to take images of the entire area of the WRIL and obtain an accurate image of the studied area, as well as of each group of pinnipeds. In September 2018, the aerial photographic census was conducted using a *DJI Mavic Pro I* UAV, flown within the same altitudinal range as during helicopter flights and with the same camera resolution.

The counts of pinnipeds were taken from the best aerial image obtained during each of the 20 flights. We used the software *Otariidae 1.0*^54^ to perform counts. Only images of days with reasonable environmental conditions that allowed for the identification of individuals on the island, in the water and around WRIL were analysed in order to avoid the impact of sea state on counting.

Individuals in the images were classified by trained researchers according to their species, age and sex by considering their morphological characteristics. For South American sea lions, five categories were considered: adult males, subadult males, juveniles, yearlings (individuals that will complete their first year of life in the same year that they were observed in the WRIL—^55^) and potential adult females. The differentiation into these categories was based on external morphology, relative size between individuals, behaviour, body colour (black in pups, turning brown with growth, and darker coloration in males in relation to females), size and shape of the head, presence, and shape and development of the mane in males^56^. Only a few individuals on the images were considered to be female in appearance (e.g. covered by yellow fur, mainly in whole head and neck, no mane and narrow muzzle in comparison to juvenile males). However, taking into account that females are rare at non-breeding sites^56^, we categorized these as “potential females”. For South American fur seals, we could distinguish adult males, but for subadults, juveniles and yearlings it is too difficult to attribute sex from aerial images, thus they were categorised only by age class, with sex undetermined. The age and sex classes were based on the descriptions in a review study of the species^12^.

### Temporal differences in abundance

The pinnipeds counted in all 20 flights were compared by month from 2010 to 2018, and abundances of each species were plotted. To assess monthly differences in the numbers of individuals in the WRIL during the study, we used a Generalized Linear Mixed Model with Negative Binomial error distribution for each species. We designated months as fixed effects, and year as a random effect to control for potential temporal autocorrelation within years. We then performed a Likelihood ratio test between this model and an intercept-only model with year random effects to assess the importance of months as an explanatory variable. We used the lme4 package^57^ within R 3.6.0 (www.R-project.org)^58^ for the analysis. In this way, it was possible to test the hypotheses that there would be differences in the mean abundance of pinnipeds between months due to their seasonal movements, and differences between the years sampled.

### Spatial differences in abundance

In order to test if the spatial distribution of pinnipeds in the WRIL was non-random due to possible differences in habitat preference by different species, 20 aerial images were georeferenced in ArcGis 10.6.1. This first procedure was performed using the ESRI satellite image provided by Esri^59^, using points known to be rocks and easily identifiable surfaces, as well as the island's geological boundaries. The general image of the island was also digitally divided into three sectors (North, Center and South), of equal area (~ 10,000 m^2^). To better understand and visualize the area each aerial image was overlaid with a 5 × 5 m grid layer. This grid size was chosen based on the body size of adult individuals, which can reach lengths of up to 2 m and 2.8 m for South American fur seals and South American sea lions^60^, respectively. Secondly, each individual pinniped was represented by a point in all 20 images.

Subsequently, we performed a Kernel Density Estimation (KDE)^61^ in order to: (1) estimate the density of specimens (points), (2) test whether pinnipeds in the WRIL exhibited spatial preferences, and (3) determine the percentage of territory occupied by each species or by all individuals in general. The KDE produces a continuous surface, with densities calculated at all locations (i.e. a density from which points are considered to form a cluster^62^). Therefore, the intensity estimator is a good method of evaluating the behavior of point patterns (specimens) in a given study area and is considered a very useful tool to provide an overview of the spatial distribution of a given event^63^. The annual and intra-annual variability of pinniped spatial distribution in the WRIL was investigated by comparing the monthly and annual density surfaces. For all surfaces generated by KDE, information about the size of the group was incorporated in each observation. This approach also allowed us to better explain changes in spatial distribution (i.e., expansion, shrinkage and overlap of density surfaces, as well as qualitative comparisons between the density maps prepared). KDE analyses and maps were generated for each flight using ArcGis 10.6.1, showing all sightings over the months and years analyzed. These were used to identify patterns of spatial distribution in the occupation of the WRIL by pinnipeds and to define the main areas of use by each species.

### Ethical approval

This study was based on aerial photographic census of the Wildlife Refuge of Ilha dos Lobos under the SISBIO licenses 69627 and 35606-1. Since there was no animal caught or killed during the summarized research it was conducted under the license PPECEUA05.2019 from the institutional ethics committee from Universidade do Vale do Rio dos Sinos—UNISINOS.

## Results

### Abundance and occupation of the WRIL by pinnipeds

**Figure 2:**
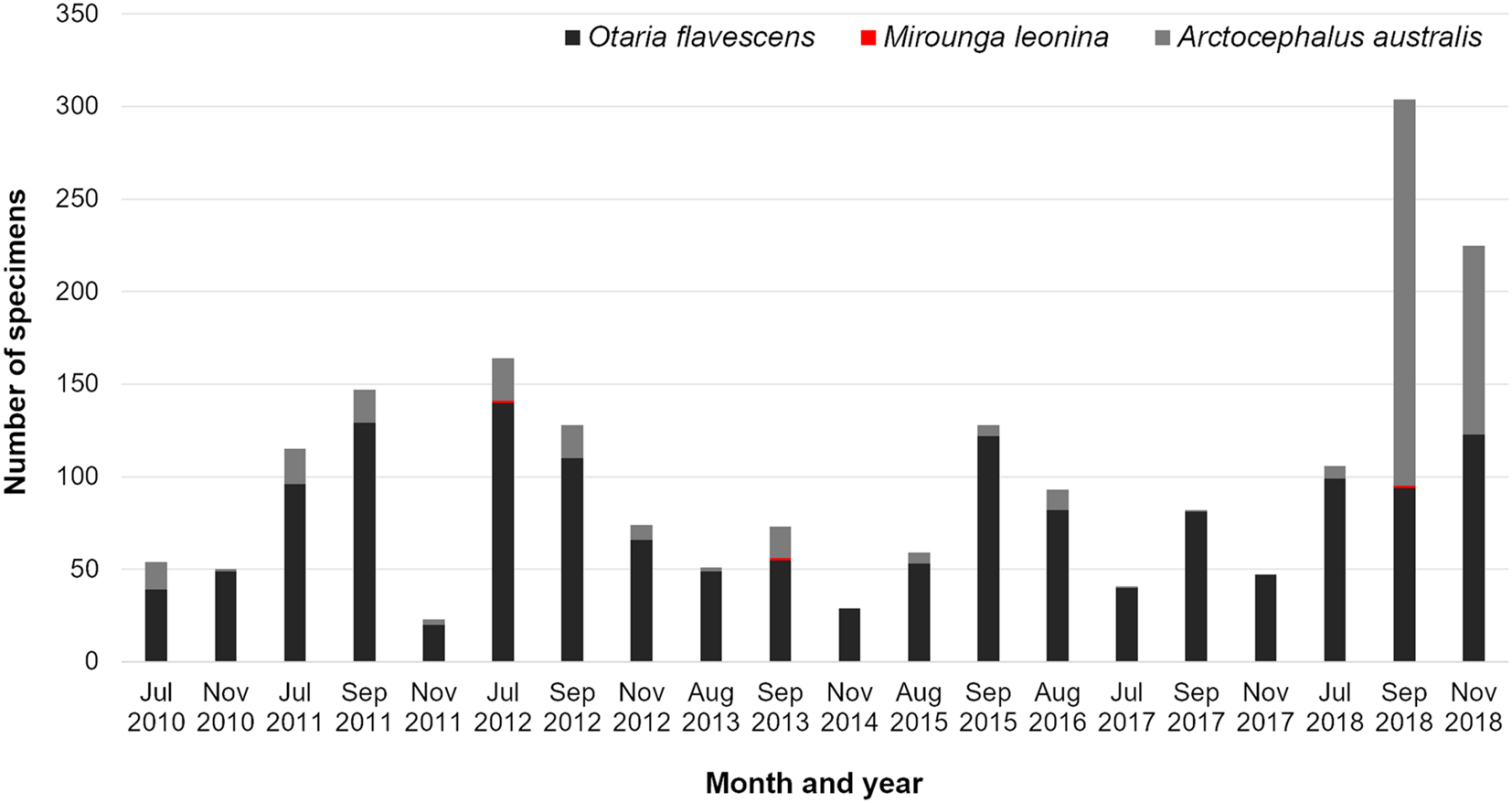
Sum of total number of pinniped individuals from three different species during seasonal occupation of the Wildlife Refuge of Ilha dos Lobos on the southern Brazilian coast (generated in excel). The data comes from 20 aerial photographic censuses from 2010 to 2018, carried out exclusively during the months July–November. Grey bar: South American fur seal (*Arctocephalus australis*) Red bar: southern elephant seal (*Mirounga leonina*), and black bar: South American sea lion (*Otaria flavescens*).

The highest monthly mean abundance of all species occupying the WRIL during the nine years of the study was found in September (mean: 143.67 SD: ± 34.16), followed by July (mean: 96 SD: ± 22.22), November (mean: 74.67 SD: ± 30.95), and August (mean: 67.67 SD: ± 12.88). Between 2010 and 2018, September 2018 presented the highest abundance of pinnipeds with 304 individuals occupying the island simultaneously, including the three species: *A. australis*, *O. flavescens* and *M. leonina*. The second month with the highest abundance of pinnipeds was November 2018, with 225 individuals occupying the island (Fig. 2). Notwithstanding the atypically high abundance in November 2018, September and July were the months when census numbers were generally the highest (Fig. 2).

The abundance of the pinnipeds in the WRIL was best explained by the intercept-only model which performed better than the model that included month as a predictor of abundance for both South American sea lions (χ^2^ = 5.77, df = 3, p = 0.12) and South American fur seals (χ^2^ = 5.32, df = 3, p = 0.15; Table 1). Therefore, there is no statistical difference in the average abundances between these months for both species, indicating that the month the surveys were carried out (i.e. July–November) had no effect on pinniped abundance on the island. The standard deviation of the estimated among-year variation was 0.18 individuals for South American sea lions and 1.32 individuals for South American fur seals.

**Table 1:** Model results based on the Negative Binomial GLMM model for pinniped counts.

Species	Parameter	Estimate	Standard Error	*z*-value	*p*-value
South American sea lion	Intercept	4.11	0.25	16.45	< 2e−16
	July	0.24	0.32	0.76	0.45
	September	− 0.14	0.31	− 0.45	0.65
	November	0.45	0.29	1. 51	0.13
South American fur seal	Intercept	1.68	0.84	2.00	0.04
	July	0.65	0.96	0.67	0.50
	September	− 0.39	0.94	− 0.41	0.68
	November	0.88	0.83	1.06	0.30

Model structure included month as a fixed effect and year as a random effect. The intercept corresponds to the month of August.

**Figure 3:**
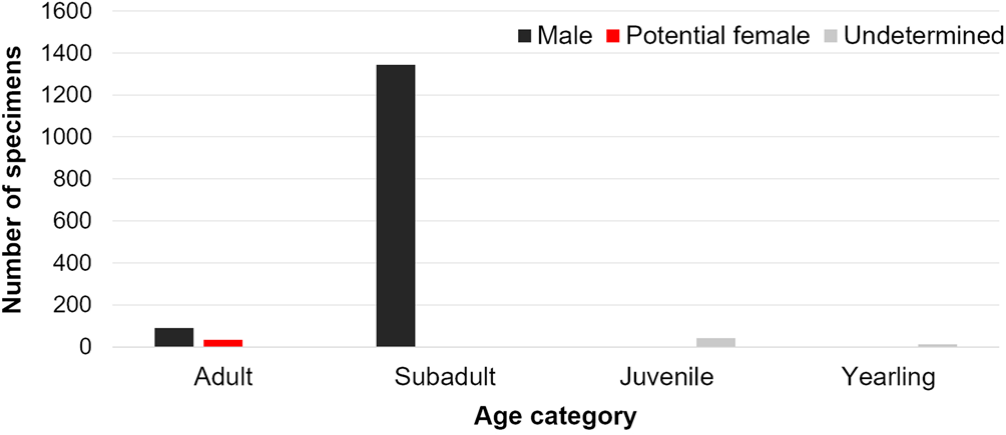
Sum of South American sea lion (*Otaria flavescens*) individuals of all age categories of that occupied the Wildlife Refuge of Ilha dos Lobos on the southern Brazilian coast (generated in excel) on the data comes from 20 aerial photographic censuses from 2010 to 2018 carried out exclusively during the months July–November. Black bar: males; red bar: potential females; and grey bar: undetermined sex.

The South American sea lion was the most frequent and abundant species recorded on the WRIL during the study period (Fig. 2), occurring in all sampled months, but with a peak in abundance of 139 individuals in July 2012 (Fig. 2). Counts of the different sex and age categories of South American sea lions revealed that there was a predominance of subadult males (n = 1343, 88.24%), followed by adult males (n = 91, 5.98%) over the years (see Fig. 3). There were relatively few records of potential females (n = 33, 2.17%), juveniles (n = 42, 2.76%), and yearlings (n = 13, 0.85%). However, potential females were recorded during half of the surveys, while the yearlings were present only during three censuses: July 2011, and September and November 2012.

**Figure 4:**
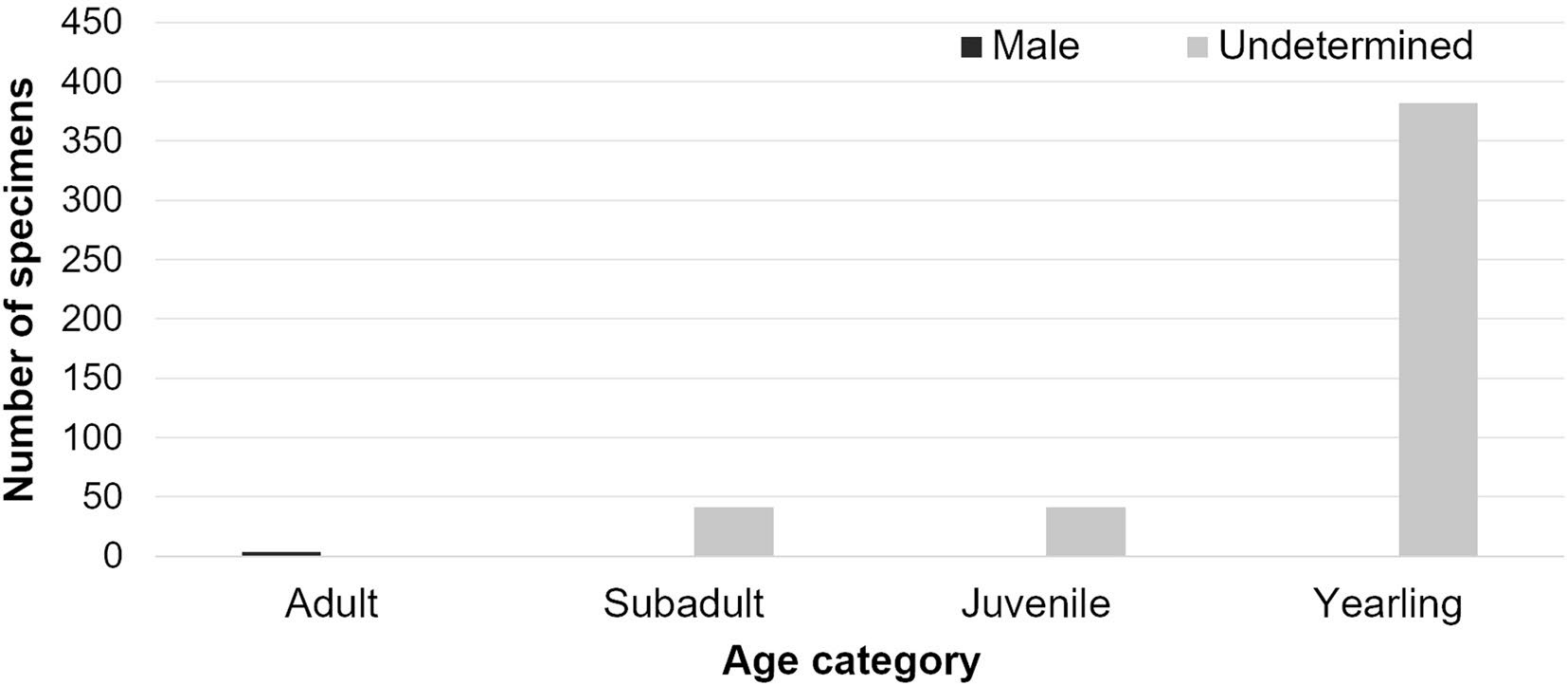
Sum of South American fur seal (*Arctocephalus australis*) individuals of all age categories of that occupied the Wildlife Refuge of Ilha dos Lobos on the southern Brazilian coast (generated in excel). The data comes from on 20 aerial photographic censuses from 2010 to 2018 carried out exclusively during the months July–November. Black bar: males and grey bar: undetermined sex.

The South American fur seal was always present in smaller numbers than South American sea lions, being the second most abundant species overall in the WRIL (n = 468), with the exception of September and November 2018 when it was the most abundant. This species was not present continually on the island, being absent in a few of the months sampled (Fig. 2), but with an unexpected peak in abundance in 2018, with 199 and 102 yearlings present in September and November of that year respectively (Fig. 2). Yearlings were the predominant age category (n = 382, 81.62%), followed by the juveniles (n = 41, 8.76%), subadults (n = 41, 8.76%), and adult males (n = 4, 0.86%) (Fig. 4). There were records of adult males in only four censuses: July and September 2011, September 2012 and July 2018.

Southern elephant seals were recorded in three censuses: July 2012, September 2013 and September 2018 (Fig. 2). The three single records were of subadult males and were identified based on body size and development of the proboscis^64^.

### Spatial occupation of WRIL by pinnipeds

**Figure 5:**
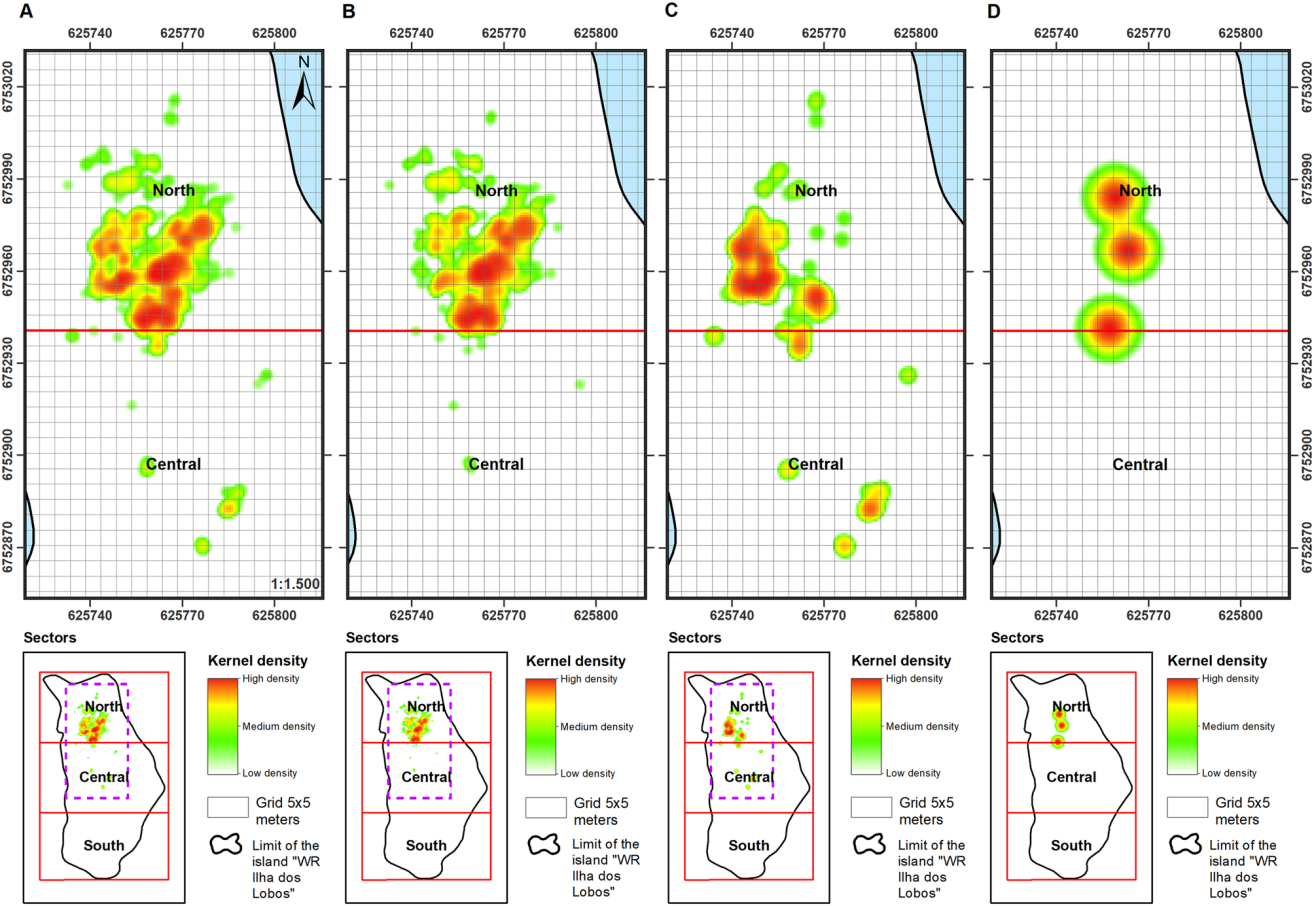
Spatial occupation of the Wildlife Refuge of Ilha dos Lobos by pinnipeds estimated using KDE for the 20 months surveyed (generated in ArcMap 10.6.1). (**A**) All three pinniped species. (**B**) South American sea lion (*Otaria flavescens*), (**C**) South American fur seal (*Arctocephalus australis*) and (**D**) Southern elephant seal (*Mirounga leonina*)*.* The emerged area of the island is present in the bottom part of the figure and the sectors analysed (north, central and south) are shown in red.

A total of 1993 animal positions in the WRIL were plotted during the 20 surveyed months, representing 1522 (76.37%) South American sea lions, 468 (23.48%) South American fur seals and three (0.15%) southern elephant seals. Of these records, 95.94% (n = 1912) were in the north zone, only 4.06% (n = 81) in the centre zone, and there were no records for the south zone (Table 2). A similar spatial occupation pattern was observed for each species individually (Table 2 and Fig. 5A–D).

**Table 2:** Summary of the spatial distribution of the cumulative records of pinnipeds across the three zones (North, Center, South) of the Wildlife Refuge of Ilha dos Lobos, southern Brazil, from 2010 and 2018.

Taxa	Number of records	North	Center	South
Pinnipeds	1993	1912 (95.94%)	81 (4.06%)	–
South American sea lions	1522	1509 (99.15%)	13 (0.85%)	–
South American fur seals	468	400 (85.47%)	68 (14.53%)	–
Southern elephant seals	3	3 (100%)	–	–

**Figure 6:**
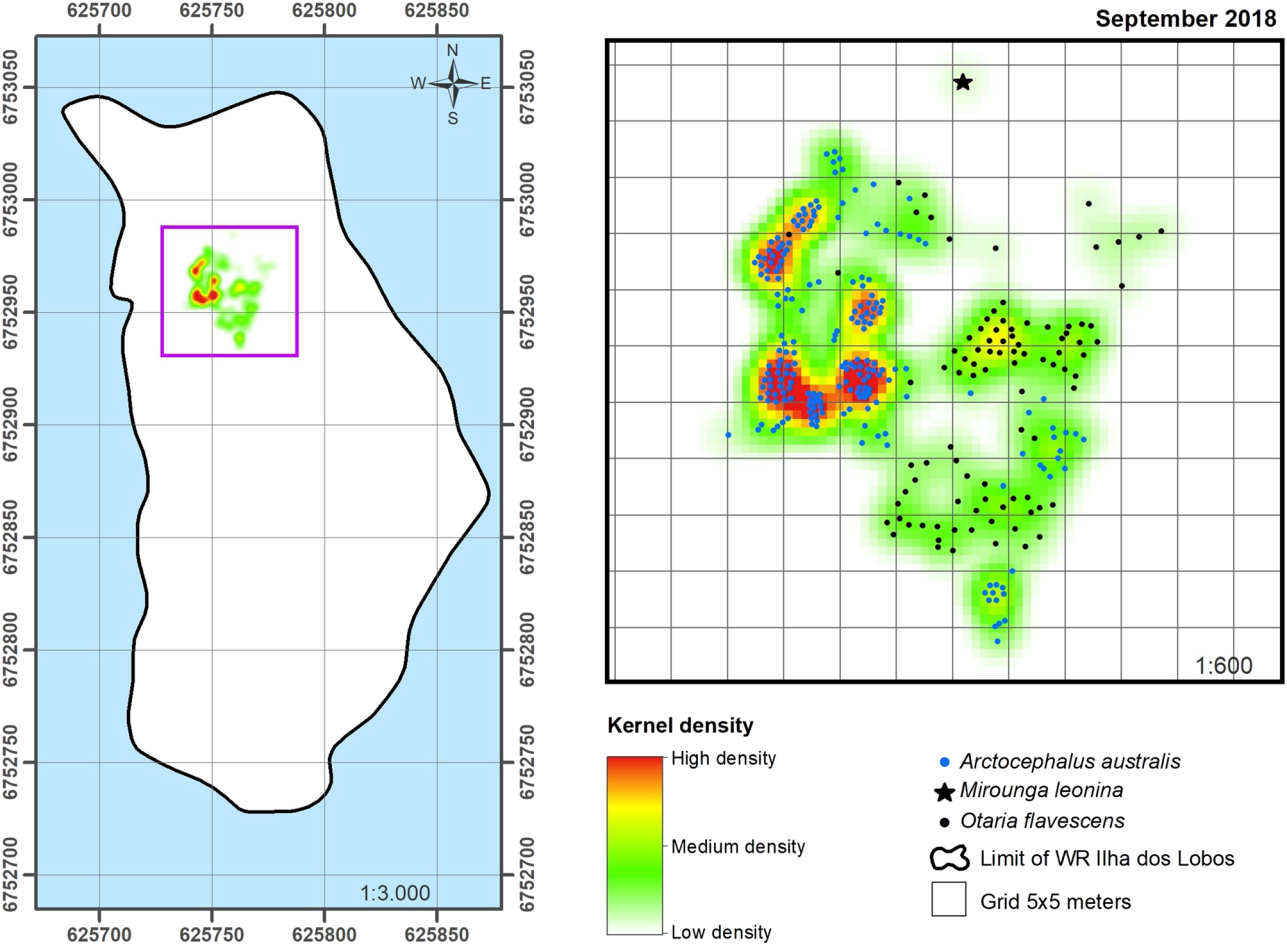
Spatial occupation on the Wildlife Refuge of Ilha dos Lobos by pinniped species for September 2018 with the KDE (generated in ArcMap 10.6.1). Blue dots: South American fur seal (*Arctocephalus australis*); black dots: South American sea lion (*Otaria flavescens*) and black star: Southern elephant seal (*Mirounga leonine*). The map shows the limit of the emerged area of the WRIL and the purple square shows the main area occupied by pinnipeds.

**Figure 7:**
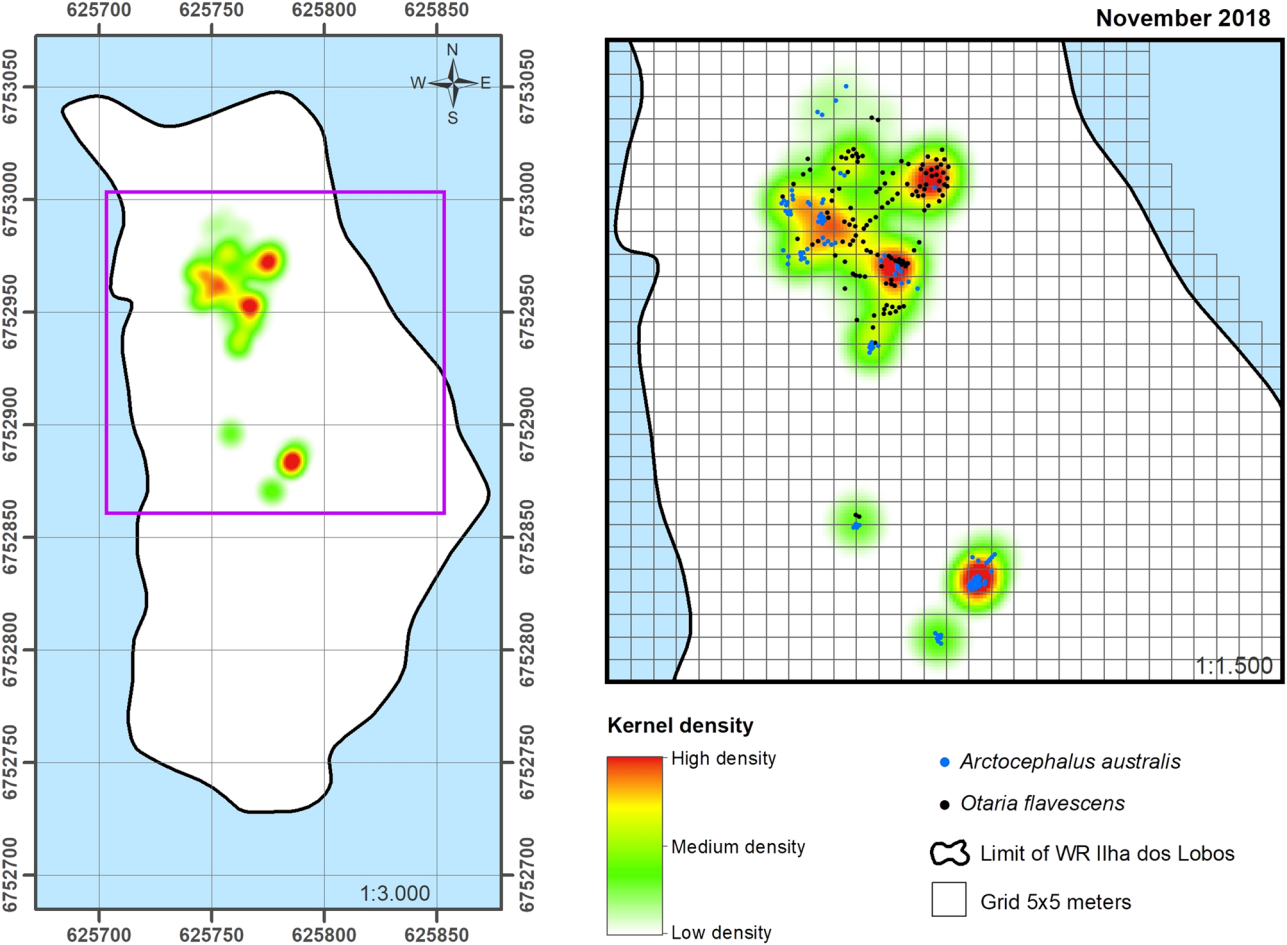
Spatial occupation of the Wildlife Refuge of Ilha dos Lobos by pinnipeds for November 2018 with the KDE (generated in ArcMap 10.6.1). The map shows the limit of the emerged area of WRIL and the purple square shows the expansion of the occupation by pinnipeds up to the central portion of the island. Blue dots: South American fur seal (*Arctocephalus australis*); black dots: South American sea lion (*Otaria flavescens*).

This uneven pattern of spatial occupation of the island by pinnipeds was also revealed by the maps showing the KDE results (Fig. 5). The pinniped density for the northern portion of the WRIL was always higher than that of the central zone of the island, and density varied between months (Table 3). The pinnipeds appeared to form clusters, mainly at the northern end of the island (Fig. 5A) where densities of the most prominent species were highest (Fig. 5B,C), and southern elephant seals were also occurred (Fig. 5D). This spatial pattern was evident in most of the sampled months, with no differences observed between the months with the highest and lowest abundances. The exception to this was September and November 2018 when the distribution extended up to the central portion of the WRIL (see Figs. 6 and 7). Pinniped densities were higher during the spring months than the winter months (Table 3), but there was no statistical difference between seasons.

**Table 3:** Pinniped density for the North and Central portions of the Wildlife Refuge of Ilha dos Lobos, southern Brazil, from 2010 and 2018.

Year	Month	Total number of individuals	North (m^2^)	Center (m^2^)
2010	July	54	0.0053	0.0001
2010	November	50	0.005	0
2011	July	115	0.0115	0
2011	September	147	0.0146	0.0001
2011	November	23	0.0023	0
2012	July	164	0.0158	0.0006
2012	September	128	0.0124	0.0004
2012	November	74	0.0074	0
2013	August	51	0.0051	0
2013	September	73	0.0073	0
2014	November	29	0.0029	0
2015	August	59	0.0058	0.0001
2015	September	134	0.0134	0
2016	August	93	0.0093	0
2017	July	41	0.004	0.0001
2017	September	82	0.008	0.0002
2017	November	47	0.0047	0
2018	July	106	0.0105	0.0001
2018	September	304	0.0292	0.0012
2018	November	225	0.0173	0.0052

Density expressed as individuals/m^2^.

**Figure 8:**
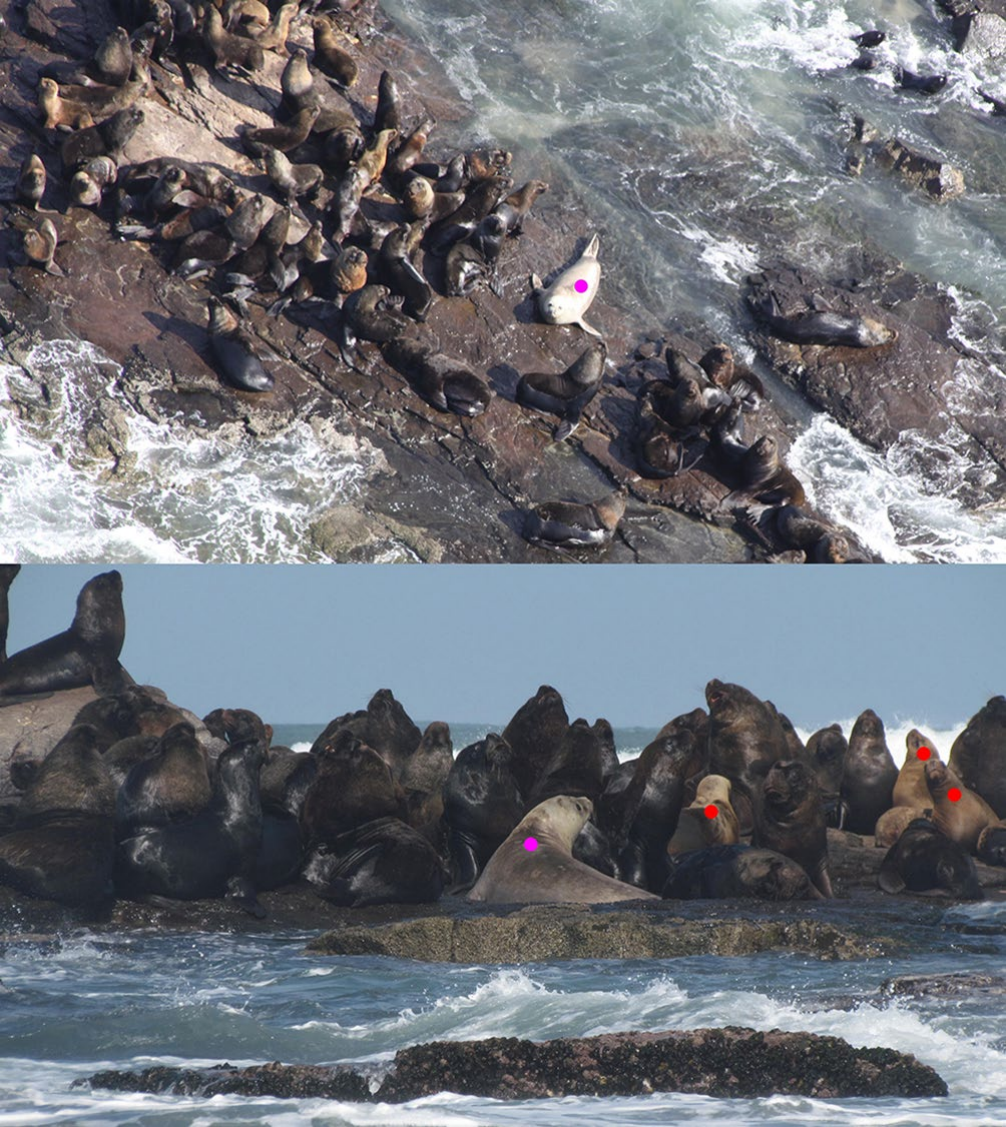
Above: aerial view of large clusters of South American sea lion specimens (*Otaria flavescens*) and one southern elephant seal (*Mirounga leonina*) (purple circle). Below: lateral view of the pinnipeds in the same day, including potential females of South American sea lions with red circles, and the southern elephant seal with purple circle, on the Wildlife Refuge of Ilha dos Lobos in July 2012.

Clusters of South American sea lions occurred in all sampled months, and included potential females (Fig. 8). The distribution of South American fur seals was more dispersed and group size was smaller than for sea lions. The fur seals tended to lie upon prominent rocks, or to surround the main groups of sea lions. Only in September and November of 2018 were the fur seals present in relatively high numbers, dispersed throughout the northern and central parts of the island (Figs. 6 and 7), with the presence of large clusters formed by yearlings (see all KDE maps for the 20 months analysed in the Online Resource). It is important to mention that no specific response by the sea lions or fur seals to the helicopter or to the UAV was observed, probably because the altitude of 100–250 m was enough to neutralize any potential reaction to the aircrafts used in this study.

## Discussion

The presence of pinnipeds in the WRIL is typically seasonal, with great concentrations in the winter and spring months, and only few records in summer and autumn^16,40^. However, this study does not confirm the further hypothesis of monthly differences in abundance during the winter and spring months. On the other hand, our data confirms the hypotheses of male bias in abundance for both species and a difference in the spatial occupation of the WRIL by South American fur seals and sea lions, with occupation mainly occurring on the northern portion of the island. Although the name “Ilha dos Lobos” means “fur seal island” in English, the most abundant pinnipeds identified during the years studied were subadults and adults of South American sea lions. In addition, yearlings of South American fur seals were also observed and occasionally there were rare observations of subadult males of southern elephant seals.

Although the seasonal abundance of pinnipeds in the WRIL was not statistically different between the spring and winter months, we found consistently high numbers in September during the years studied (except for 2012 when numbers were highest in July). This was also reported by two other previous studies^16,40^ that counted a high numbers of South American sea lions (n = 102) at Ilha dos Lobos in September of 1994. Both previous studies conducted a systematic census during a complete year, reporting the greatest concentrations of individuals between July and September, a pattern also observed in the nonbreeding site Las Pipas, in Uruguay (which is ~ 1000 km far away from the WRIL)^65^ as well as in the present study. The arrival and presence of South American fur seals and sea lions in the WRIL correspond to the dispersal period after the breeding season, which is the least known period of the life cycle of these species^15,16^. These “seasonal movements” performed by these South American pinnipeds, probably from their closest reproductive colonies in Uruguay and Argentina^2,45^ are likely assisted by the cold Malvinas Current, which is very intense during winter and spring months on the southern Brazilian coast^1,66^. The seasonal dispersal of South American sea lions was previously described for their other haulout site, the Wildlife Refuge of Molhe Leste de São José do Norte (WRML)^2^, which is located 400 km south of our study area. This southern haulout site represents the first arrival and final stopping point of the species in Brazilian waters^2^. South American sea lions mainly arrived at the WRML in the months of March and December, its occupation being slightly out of phase with the WRIL^40^. Therefore, the decreased numbers of sea lions observed in the WRIL in November, relative to September (excepting 2018) is likely associated with the southwards return of sea lions from the WRIL to the WRML, where the number of pinnipeds remains high until the end of December^10,40^. After this period, these pinnipeds returned to their breeding colonies in Uruguay, and perhaps Argentina, to start the next breeding season^2,17,65^.

In regards to species abundance in the WRIL, the numbers of South American fur seals were usually lower than numbers of South American sea lions. Low numbers of South American fur seals were observed in all censuses other than for September and November 2018, when 199 and 102 yearlings were observed respectively. In previous studies, there were records of only four South American fur seals in August 1994^16^, and 17 individuals in July 2002 for the WRIL^40^. Although the difference in abundance between two species in the WRIL is evident, it was never discussed in depth by previous studies^16,40^. However, it seems that the role played by the WRIL is different for both species to some extent. Although both species seem to take advantage of this area of lower human disturbance, mainly compared to the beaches nearby, for resting, foraging^67,68^ and for the control of thermal stress^16^, the geographical position of the island, the predominant age category of each species in Brazilian waters and their ecological niche may explain some of the differences found.

The South American sea lion is an opportunistic predator that consumes different prey species according to their availability in the environment^67^. In Southern Brazil, there are signs that the species has changed its feeding habits over the last decades due to changes in the abundance of different fish stocks in the area^69^. Moreover, the species is well-known for its behavior of following fishing boats and catching fish from the nets^69–71^. Therefore, the position of the WRIL close to an important fishing harbour (Torres/Passo de Torres—see^72^ for a description of the fisheries), may be advantageous for the species. In contrast, the feeding habits of South American fur seals are poorly known in the region. A local feeding ecology study indicated that most South American fur seals examined presented empty stomachs, while sea lion stomachs are usually full. This may suggest an offshore foraging behaviour or even that the fur seals did not feed properly along southern Brazilian coast^67^. Moreover, it is worth mentioning that every year hundreds of South American fur seals, mainly young and yearlings, are found dead on the beaches of southern Brazil^73–75^. Many of these individuals present with severe trauma, respiratory problems, and disorders in muscular, central nervous and hepatic systems as well as in the gastrointestinal tract, with mortality mainly associated with cachexia^76^. This pattern suggests that the southern Brazilian coast could be a sink habitat^77^ for the species. A similar pattern seems to exist for the Magellanic penguin (*Spheniscus magellanicus*) in the region. Every year, mainly during the winter and spring months (July–September), thousands of Magellanic penguins are found dead ashore on the Brazilian coast (e.g. Ref.^78^). The closest breeding colonies of these marine birds to our study site are located in Argentine Patagonia and their presence on the Brazilian coast is largely influenced by the Malvinas Current^78,79^. Interestingly, the overwhelming majority of these marine birds are first-year juveniles and present a relatively poor body condition^80,81^; a very similar pattern to the South American fur seals.

Nevertheless, the increased abundance of South American fur seals at the end of the time series of the present study (September and November 2018) is a tricky result to explain, but could relate to population growth or natural fluctuations in births and survival from year to year^29^. Non-published beach survey data for the same period and region also confirmed the unusually high numbers of South American fur seals from August to September 2018 (M. Tavares pers. comm.). However, additional monitoring, not only in the WRIL but also in areas further north on the Brazilian coast, is required to determine if the 2018 peaks were an anomaly or if they were related to population growth in their breeding colonies, or even range expansion.

The scenario for South American sea lions is very different to that for South American fur seals, with number across the years staying relatively constant in the WRIL. Moreover, the mortality of the species in the region over the last decades is relatively low compared to South American fur seals and most South American sea lions found dead are apparently healthy but show evidence of human-induced mortality^82,83^. Regarding the sex and age classes of South American sea lions found in the WRIL, the relatively high abundance of subadult and adult males was also reported by beach surveys along the coast of Rio Grande do Sul^1–3,73^, or in the WRIL^16,40^. Despite the predominance of males reported during the study period, we also observed potential female South American sea lions in 10 out of 20 flights, and females had previously only been briefly mentioned for the WRIL in 1995^3^. More than a dozen adult female South American sea lions^73,84^ and four pregnant female South American fur seals^85^ were also found dead or stranded along the same coastline. This difference in abundance of females and males occupying the WRIL is probably related to the fact that males do not provide parental care during the post-breeding period^2,11,14^. In contrast, females need to nurse their pups on land for at least eight months in the reproductive colonies^11,60^. These potential adult females observed in the WRIL in the present study had probably either skipped breeding for the year, or had lost their pup during the lactation period, and thus they were able to disperse as they were not tied to a breeding colony.

The presence of yearling South American sea lions in the WRIL was recorded during three months of this study and was also previously reported by the local fishermen during interviews in 2013^71^. However, the scientific evidence in the form of photographic images were never reported until now. Although the Brazilian coast is not a breeding area for South American sea lions or South American fur seals, there were previous records of weaned pup and yearling South American sea lions in this area^2,75^. The yearlings of both species recorded in the WRIL were born in the previous breeding period (between November and February)^11,45,74^, and probably came from the nearest breeding colonies in Uruguayan waters^1,2^. They may have been taken accidentally by the cold Malvinas Current^1,2,4,16,66^ during their first sea incursions after being weaned^73^, as pups are less proficient swimmers than adults^2^.

During the nine years of this study, southern elephant seals were recorded only on three occasions in the WRIL. This species is considered less frequent on the Brazilian coast, despite several records across the country (e.g. Refs.^1,3,73–75,86–88^), including for the WRIL^40^. The individuals found at the WRIL, all of which were subadult males, were most probably from the colonies at Peninsula Valdés, on the central coast of Argentina, considering that these are the closest southern elephant seal colonies to the Brazilian coast^88,89^. These southern elephant seals reached the WRIL probably assisted by the cold Malvinas Current as for the other South American pinnipeds^1,66^.

The spatial occupation of the WRIL by pinnipeds shows a clear pattern of distribution of both of the most prominent species. South American sea lions preferred to stay in groups in the northern portion of the island. These sea lions show a positive thigmotaxia, which is a tendency in some pinnipeds to cluster tightly together on land^49^, in order to maintain body contact^90^. In some cases, individuals are so tightly clumped that they are literally “piled” on top of each other; this occurs mainly on the flat areas of the WRIL. A similar pattern was also shown by groups of yearling South American fur seals, in September 2018, possibly driven by the limited space on the island. The area preferred by the pinnipeds contains big rocks and large flat dry areas, where individuals remain resting for long periods^31^. Both species seem to prefer the northern portion of the WRIL, which apparently is higher than the rest of the island. Pinnipeds do not occupy the southern regions even during low tide when they are uncovered by water. This concentration of fur seals and sea lions in the northern area of the WRIL is probably because it is the driest and most protected environment on the island during high tides, providing the animals shelter against the constant waves. Previous authors^16,40^ have briefly mentioned this northern area as a concentration zone for most of the pinnipeds on the island, but without providing any systematic analysis or detailed information of the positions or preferences by species on the WRIL.

The present results allow us to understand more clearly where and when pinnipeds use the WRIL. This information on monthly abundance and spatial occupation may assist future management and conservation plans both for pinnipeds and for this MPA as a whole. The WRIL and its surroundings are subject to intense anthropogenic activity, resulting from tourism and fishing activities in the region^47^. In this context, we suggest that potential activities on the WRIL, such as land research and recreational activities around the island, must occur preferentially in the summer months when pinnipeds are largely absent. On the other hand, during the winter and spring months, marine mammal-based tourism may be encouraged and formally regulated, since it is the main period of occupation of the islands by pinnipeds, accompanied by the presence of southern right whales in the region^53^. Nevertheless, considering that the island is an MPA, such activities should be controlled and combined with environmental education in order to promote positive results for the conservation of the WRIL.

It is important to mention that currently the WRIL has neither a buffer zone nor a management plan, compromising any effective monitoring of human activities as well as the evaluation of their impacts. Our results could assist with the delineation of management zones in future management plans for the MPA, taking into account the seasonal and spatial occupation of the island by pinnipeds. In this context, we suggest the introduction of a “flexible restrictive zone”, which could vary in size according to the use of the MPA by pinnipeds and the season, being larger and more restrictive for some activities during the winter and spring months to accommodate the area occupied by pinnipeds at this time and avoiding disturbing them. Moreover, we also recommend behaviour response studies related to the distance that tourist boats could operate during the winter and spring months, which correspond to the seasons in which pinnipeds are present in the WRIL, in order to establish the minimum approach distance to the island.

Finally, our data suggest that the WRIL can potentially be used as a control area to detect oscillations or populations trends in the two main pinniped species (i.e. South American sea lions and South American fur seals) which occur there, at least for populations in their closest breeding sites. Despite the limitation of the island in terms of available area, its biogeographical location at the border of both species’ distributions may mean it is sensitive to population changes. To explore this further, ideally the WRIL should be included in a network of monitoring sites, covering at least the main breeding colonies in Uruguay during the same time-series and definitive links to breeding areas should be established (Supplementary information S1).

## Supplementary information

Supplementary Information

## References

[1] Pinedo, M.C. Ocorrência de pinípedes na costa brasileira. *Garcia de Orta Serie de Zoologia* **15**, 37–48 (1990).

[2] Rosas FCW, Pinedo MC, Marmotel M, Haimovici M (1994). Seasonal movements of the South American sea lion (*Otaria flavescens* Shaw, 1800) of the Rio Grande do Sul coast Brazil. Mammalia.

[3] Simões-Lopes PC, Drehmer CJ, Ott PH (1995). Nota sobre os Otariidae e Phocidae (Mammalia: Carnivora) da costa norte do Rio Grande do Sul e Santa Catarina Brasil. Biociências.

[4] Oliveira, L.R. Carnívoros marinhos in *Mamíferos do Rio Grande do Sul* (eds. Weber, M.M., Roman, C. & Cáceres, N.C.) 405-227 (Editora UFSM, 2013).

[5] Oliveira, L.R., Danilewicz, D., Martins, M.B., Ott, P.H., Moreno, I.B., Caon, G. New records of the Antarctic fur seal, *Arctocephalus gazella* (Peters, 1875) to the Brazilian coast. *Com. Museu de Ciência e Tecnologia da PUCRS* **14**, 201–207 (2001).

[6] Oliveira LR, Machado R, Alievi MM, Würdig NL (2006). Crabeater seal (*Lobodon carcinophaga*) on the coast of Rio Grande do Sul State, Brazil. LAJAM.

[7] Frainer G, Heissler VL, Moreno IB (2017). A wandering Weddell seal (*Leptonychotes weddellii*) at Trindade Island, Brazil: the extreme sighting of a circumpolar species. Polar Biol..

[8] Milmann, L., Machado, R., Oliveira, L.R., Ott, P.H. Far away from home: presence of fur seal (*Arcocephalus* sp.) in the equatorial Atlantic Ocean. *Polar Biol.* **42**, 817–822 (2019).

[9] Rocha-Campos, C.C., Câmara, I.G. *Plano de ação nacional para conservação dos mamíferos aquáticos: grandes cetáceos e pinípedes. Instituto Chico Mendes de Conservação da Biodiversidade.* 156 (ICMBio, 2011). https://www.icmbio.gov.br/cma/images/stories/pans_grandes_cetaceos_e_pinipedes/Pequenos_cet%C3%A1ceos_PAN.pdf.

[10] Pavanato H, Silva KG, Estima SC, Monteiro DS, Kinas PG (2013). Occupancy dynamics of South American sea lions in Brazilian Haul-outs. Braz. J. Biol..

[11] Campagna C (1985). The breeding cycle of the southern sea lion, *Otaria byronia*. Mar. Mammal Sci..

[12] Vaz-Ferreira R (1982). *Arctocephalus australis* (Zimmermann): South American fur seal. Mammals Seas FAO Fish. Ser..

[13] Francu-Treco V, Costa P, Scharam Y, Tassino B, Inchausti P (2014). Sex on the rocks: reproductive tactics and breeding success of South American fur seal males. Behav. Ecol..

[14] Campagna C (2001). Movements and location at sea of South American sea lions (*Otaria flavescens*). J. Zool..

[15] Bastida, R. & Rodríguez, D. Hallazgo de un apostadero estacional de lobos marinos de dos pelos, *Arctocephalus australis* (Zimmermann, 1783), en bajos fondos frente a la costa de Mar del Plata (Provincia de Buenos Aires, Argentina). *Anales 4ª Reunión de Trabajo de Especialistas en Mamíferos Acuáticos de América del Sur* 1–22 (1994).

[16] Sanfelice D, Vasques VC, Crespo EA (1999). Ocupação sazonal por duas espécies de Otariidae (Mammalia, Carnivora) da Reserva Ecológica Ilha dos Lobos, Rio Grande do Sul Brasil. Iheringia, Sér. Zool..

[17] Giardino GV (2014). Travel for sex: Long-range breeding dispersal and winter haulout fidelity in Southern sea lion males. Mammal. Biol..

[18] Oliveira LR (2008). Morphological and genetic evidence for two evolutionarily significant units (ESUS) in the South American fur seal *Arctocephalus australis*. Conserv. Genet..

[19] Oliveira LR (2017). Ancient female philopatry, asymmetric male gene flow, and synchronous population expansion support the influence of climatic oscillations on the evolution of South American sea lion (*Otaria flavescens*). PLoS ONE.

[20] Cárdenas-Alayza, S., Crespo, E.A., Oliveira, L., R. *Otaria byronia*. The IUCN Red List of Threatened Species 2016: e.T41665A61948292. https://doi.org/10.2305/IUCN.UK.2016-1.RLTS.T41665A61948292.en (2016).

[21] Páez, E. Situación de la administración del recurso lobos y leones marinos en Uruguay in *Bases para la conservación y el manejo de la costa Uruguaya* (eds. Menafra, R., Rodríguez-Gallego, L., Scarabino, F., Conde, D. 577–583 (Sociedad Uruguaya para la Conservación de la Naturaleza, Montevideo 2006).

[22] Franco-Trecu, V. Tácticas comportamentales de forrajeo y apareamiento y dinámica poblacional de dos especies de otáridos simpátricas con tendencias poblacionales contrastantes. PhD Thesis. Universidad de la República (UdelaR) Montevideo, Uruguay (2015). https://hdl.handle.net/20.500.12008/6895.

[23] Crespo EA, Oliva D, Dans S, Sepúlveda M (2012). Estado de situación del lobo marino común en su área de distribución.

[24] Baylis AMM (2015). Disentangling the cause of a catastrophic population decline in a large marine mammal. Ecology.

[25] Cárdenas-Alayza, S., Oliveira, L.R., Crespo, E.A. *Arctocephalus australis*. The IUCN Red List of Threatened Species 2016: e.T2055A45223529. https:// doi.org/10.2305/IUCN.UK.2016-1.RLTS.T2055A45223529.en. (2016).

[26] Baylis AMM (2019). Re-evaluating the population size of South American fur seals and conservation implications. Aquat. Conserv. Mar. Freshw. Ecosyst..

[27] Harwood J, Prime JH (1978). Some Factors affecting the size of British grey seal populations. J. Appl. Ecol..

[28] Páez, E. Utilización de Boostrap y analisis de poder en estimaciones de abundancia de cachorros de *Arctocephalus australis* [Using Bootstrap and power analysis in abundance estimates of *Arctocephalus australis* pups] in *Sinopsis de la Biologıa y Ecologıa de las Poblaciones de Lobos Finos y Leones Marinos de Uruguay [Synopsis of the biology and ecology of populations of fur seals and sea lions of Uruguay]* (eds. Rey, M., Amestoy, F.) 55–70 (Proyecto URU/92/003, INAPE, Montevideo, Uruguay, 2000).

[29] Franco-Trecu V (2019). Abundance and population trends of the South American Fur Seal (*Arctocephalus australis*) in Uruguay. Aquat. Mammals.

[30] Crespo, E.A. & Oliveira, L.R. South American fur seal (*Arctocephalus australis*, Zimmerman 1783) in *Ecology and Conservation of Pinnipeds in Latin America* (eds. Heckel, G., Schramm, Y.) (Springer Nature, *in press*).

[31] Sanfelice D, Vasques VC, Romanowski HP, Cappozzo HL (2015). Activity budget in South American Sea Lions (*Otaria flavescens*) in the most northern South-Atlantic haul-out site. Bol. Soc. Bras. Mastozool..

[32] McIntosh RR (2018). Understanding meta-population trends of the Australian fur seal, with insights for adaptive monitoring. PLoS ONE.

[33] Eberhardt LL, Chapman DG, Gilbert JR (1979). A review of marine mammal census methods. Wildl. Monogr..

[34] Forney, K.A. Surveys in *Encyclopedia of Marine Mammals* (eds. Perrin, W.F., Wursig, B., Thewissen, J.G.M.) 129–1131 (Academic Press, 2009).

[35] Grandi MF, Dans SL, Crespo EA (2008). Social composition and spatial distribution of colonies in an expanding population of south American sea lions. J. Mammal..

[36] Lowry, M.S., W.L. Perryman, M.S. Lynn, R.L. Westlake, F.J. Counts of northern elephant seals, *Mirounga angustirostris*, from large-format aerial photographs taken at rookeries in southern California during the breeding season. *Fish. Bull. Natl Ocean. Atmos. Admin.* **94**, 176–185 (1996).

[37] Adame K, Pardo MA, Salvadeo C, Beier E, Elorriaga-Verplancken FR (2017). Detectability and categorization of California sea lions using an unmanned aerial vehicle. Mar. Mammal Sci..

[38] Hiby AR, Thompson D, Ward AJ (1988). Census of grey seals by aerial photography. Photogram. Rec..

[39] Heide-Jorgensen MP (2004). Aerial digital photographic surveys of narwhals, *Monodon monoceros*, in northwest Greenland. Mar. Mammal Sci..

[40] Silva, K.G. Os pinípedes no Brasil: ocorrências, estimativas populacionais e conservação. PhD thesis. Fundação Universidade Federal de Rio Grande, Rio Grande (2004).

[41] Silva, K.G., Araújo, T.G., Crivellaro, C.V.L., Menezes, R.B. *Os Mamíferos Marinhos do Litoral do Rio Grande do Sul* (NEMA, 2014).

[42] Small RJ, Pendleton GW, Pitcher KW (2003). Trends in abundance of Alaska harbor seals, 1983–2001. Mar. Mammal Sci..

[43] Sepúlveda M (2011). Distribution and abundance of the South American sea lion *Otaria flavescens* (Carnivora: Otariidae) along the central coast off Chile. Rev. Chil. Hist. Nat..

[44] Li J, Heap AD (2011). A review of comparative studies of spatial interpolation methods in environmental sciences: Performance and impact factors. Ecol. Inf..

[45] Vaz-Ferreira, R. *Otaria flavescens* (Shaw): South American sea lion. Mammals in the Seas. *FAO Fisheries series* **4**, 477–495 (1982).

[46] Castilho PV, Simões-Lopes PC (2008). Sea mammals in archaeological sites on the southern coast of Brazil. Rev. Mus. Arqueol. Etnol..

[47] Engel MT, Marchini S, Pont AC, Machado R, Oliveira LR (2014). Perceptions and attitudes of stakeholders towards the Wildlife Refuge of Ilha dos Lobos, a marine protected area in Brazil. Mar. Policy.

[48] Warneke RM (1975). Dispersal and mortality of juvenile fur seals, *Arctocephalus pusillus doriferus*, in Bass Strait, Southeastern Australia. Rapports et Proces Verbaux des Reunions du Conseil International pour l’Exploration de la Mer.

[49] Riedman, M. *The Pinnipeds*. 439 (University of California Press, 1990).

[50] Brasil. Decreto no. 88.463, de 4 de julho de 1983. Cria a Reserva Ecológica Ilha dos Lobos, e dá outras providencias. *Diário Oficial da República Federativa do Brasil* **129**, 12009 (1983).

[51] Brasil. Decreto de 4 de julho de 2005. Presidência da República-Casa Civil- Subchefia para Assuntos Jurídicos. 04 de julho de 2005. https://www.planalto.gov.br/ccivil_03/_Ato2004-2006/2005/Dnn/Dnn10578.htm (2005).

[52] Groch KR, Palazzo JT, Flores PAC, Adler FR, Fabian ME (2005). Recent rapid increases in the right whale (*Eubalaena australis*) population off southern Brazil. Latin Am. J. Aquat. Mammals.

[53] Danilewicz D, Moreno IB, Tavares M, Sucunza F (2016). Southern right whales (*Eubalaena australis*) off Torres, Brazil: group characteristics, movements, and insights into the role of the Brazilian-Uruguayan wintering ground. Mammalia.

[54] Bartheld, J.L., Pavés, H., Contreras, F. Cuantificación poblacional de lobos marinos en el litoral de la I a IV Regiones. *Final report proyecto FIP 2006-50* (2008).

[55] King, J.E. *Seals of the World* (British Museum of Natural History, 1983).

[56] Crespo. E.A. Dinámica poblacional del lobo marino de un pelo *Otaria flavescens* (Shaw, 1800), en el norte del Litoral Patagónico. PhD Thesis Ciencias Biológicas Facultad de Ciencias Exactas y Naturales. Universidad Nacional de Buenos Aires, Argentina (1988). https://digital.bl.fcen.uba.ar/Download/Tesis/Tesis_2107_Crespo.pdf.

[57] Bates D, Maechler M, Bolker B, Walker S (2015). Fitting linear mixed-effects models using lme4. J. Stat. Softw..

[58] R Core Team. R: A language and environment for statistical computing. R Foundation for Statistical Computing, https://www.R-project.org/ (2019).

[59] ESRI. ArcGIS Desktop: Release 10.5 Redlands (Environmental Systems Research Institute, 2018).

[60] Bastida, R. & Rodríguez, D. *Mamíferos marinos de Patagonia y Antártida* (Editorial Vazquez Mazzini, 2003).

[61] Silverman, B.W. *Density Estimation for Statistics and Data Analysis* (Chapman and Hall, 1986).

[62] Diggle PJ (1985). A kernel method for smoothing point process data. Appl. Stat. Amsterdam.

[63] Druck, S., Carvalho, M.S., Câmara, G., Monteiro, A.V.M. *Análise Espacial de Dados Geográficos*. (EMBRAPA, 2004).

[64] Lewis M (1996). Elefante marino del sur: biología de la especie, descripción general de la agrupación de la Península Valdés y protocolos de trabajo. Informes Técnicos del Plan de Manejo Integrado de la Zona Costera Patagónica. Puerto Madryn. Argentina.

[65] Szteren D (2015). *Otaria flavescens* and *Arctocephaus australis* abundance in poorly known sites: a spatial expansion of colonies?. Braz. J. Oceanogr..

[66] Seeliger, U., Odebrecht, C., Castelo, J.P. Os ecossistemas costeiro e marinho do extremo sul do Brasil. (Ecoscientia, 1998).

[67] Oliveira, L.R., Ott, P.H., Malabarba, L.R. Ecologia alimentar dos pinípedes do sul do Brasil e uma avaliação de suas interações com atividades pesqueiras in *Ecologia de mamíferos* (eds. Reis, N.R., Peracchi, A.L., Santos, G.A.S.D.) 93–109 (Technical Books Editora, Londrina, 2008).

[68] Machado R (2020). Trophic overlap between marine mammals and fisheries in subtropical waters in the western South Atlantic. Mar. Ecol. Prog. Ser..

[69] Machado R (2008). Changes in the feeding ecology of South American sea lions on the southern Brazilian coast over the last two decades of excessive fishing exploration. Hydrobiologia.

[70] Machado R, Oliveira LR, Montealegre-Quijano S (2015). Incidental catch of South American sea lion in a pair trawl off southern Brazil. Neotropic. Biol. Conserv..

[71] Pont AC (2016). The human dimension of the conflict between fishermen and South American sea lions in southern Brazil. Hydrobiologia.

[72] Moreno IB, Danilewicz D, Tavares M, Ott PH, Machado R (2009). Descrição da pesca costeira de média escala no litoral norte do Rio Grande do Sul: comunidades pesqueiras de Imbé/Tramandaí e Passo de Torres/Torres. Boletim do Instituto de Pesca (Online).

[73] Oliveira, L. R. Caracterização dos padrões de ocorrência dos pinípedes (Carnivora: Pinnipedia) ocorrentes no litoral do Rio Grande do Sul, Brasil, entre 1993 e 1999. Master Dissertation. Pontifícia Universidade Católica do Rio Grande do Sul (PUCRS), Porto Alegre, Brasil (1999).

[74] Oliveira A (2011). Occurrence of pinnipeds in Santa Catarina between 2000 and 2010. Latin Am. J. Aquat. Mammals.

[75] Prado, J.H.F., Mattos, P.H., Silva, K.G., Secchi, E.R. Long-Term Seasonal and Interannual Patterns of Marine Mammal Strandings in Subtropical Western South Atlantic. *PLoS ONE ***11**, e.0146339 (2016). 10.1371/journal.pone.0146339 PMC4729480 26814667

[76] Baldassin, P., Armorim, D.B., Werneck, M.R. Pathologies of Pinnipeds in Brazil in *Pinnipeds Bio-Ecology, Threats and Conservation* (eds. Avalva, J.) 269–285 (Ed. Taylor & Francis Group) (2017).

[77] Pulliam HR (1988). Sources, sinks, and population regulation. Am. Nat..

[78] Dantas G (2013). Evidence for northward extension of the winter range of Magellanic penguins along the Brazilian coast. Mar. Ornithol..

[79] Marques FP, Cardoso LG, Haimovici M, Bugoni L (2018). Trophic ecology of Magellanic Penguins (*Spheniscus magellanicus*) during the non-breeding period. Estuar. Coast Shelf. Sci..

[80] Garcia-Borboroglu P (2010). Magellanic penguin mortality in 2008 along the SW Atlantic Coast. Mar. Pollut. Bull..

[81] de Paula AA (2020). Host–parasite relationship in Magellanic Penguins (*Spheniscus magellanicus*) during their long northward journey to the Brazilian coast. Polar Biol..

[82] Rosas FCW, Haimovici M, Pinedo MC (1993). Age and growth of the South American sea lion, *Otaria flavescens* (Shaw, 1800), in southern Brazil. J. Mammal..

[83] Machado, R. *et al*. Mortalidade de *Otaria flavescens* devido a interações com a atividade pesqueira no sul do Brasil in *15a Reunión de Trabajo de Expertos en Mamíferos Acuáticos de América del Sur y 9º Congreso de la Sociedad Latino Americana de Especialistas en Mamíferos Acuáticos (SOLAMAC),* Puerto Madryn (2012).

[84] Drehmer, C.J. Variação geográfica em *Otaria byronia* (de Blainville, 1820) (Pinnipedia, Otariidae) com base na morfometria sincraniana. PhD thesis, Universidade Federal do Rio Grande do Sul, Porto Alegre (2005). https://hdl.handle.net/10183/8135.

[85] Muelbert MMC, Oliveira LR (2006). First records of stranded pregnant female South American fur seals, *Arctocephalus australis*, in the southern Brazilian cost. LAJAM.

[86] Castello HP, Pinedo MC (1977). Os visitantes ocasionais de nosso litoral. Natureza em Revista.

[87] Lodi L, Siciliano S (1989). A southern elephant seal in Brazil. Mar. Mammal Sci..

[88] Moura J, Di Dario B, Lima L, Siciliano S (2010). Southern elephant seals (*Mirounga leonina*) along the Brazilian coast: review and additional records. Mar. Biodivers. Rec..

[89] Lewis M, Campagna C, Marin MR, Fernandez T (2006). Southern elephant seals north of the Antarctic Polar Front. Antarct. Sci..

[90] Kirkwood, R. & Goldsworthy, S. *Fur seals and sea lions*. (CSIRO Publishing, 2013).

