# Supplementary material for: The northernmost haulout site of South American sea lions and fur seals in the western South Atlantic
Source: Sci Rep. 2020 Nov 17;10:20008. doi: 10.1038/s41598-020-76755-2 (PMC7672111; doi:10.1038/s41598-020-76755-2)
Supplement: Supplementary file 1 — Supplementary Information [file 41598_2020_76755_MOESM1_ESM.docx]

**Supplementary material**

The northernmost haulout site of South American sea lions and fur seals in the western South Atlantic

Running Title: Northernmost haulout site for pinnipeds in the western South Atlantic

Natália Procksch^1^, M. Florencia Grandi^2^, Paulo Henrique Ott^3,4^, Karina Groch^5^, Paulo A.C. Flores^6^, Marcelo Zagonel^7,8^, Maurício Veronez^8^, Enrique A. Crespo^2^, Rodrigo Machado^4^, Guido Pavez^9^, Murilo Guimarães^10^ & Larissa Rosa de Oliveira^1,4,*^

^1^ Laboratório de Ecologia de Mamíferos (LEM), Universidade do Vale do Rio dos Sinos (UNISINOS), Av. Unisinos 950, Cristo Rei, São Leopoldo, RS 93022-750, Brazil.

^2^ Laboratório de Mamíferos Marinos, Centro para el Estudio de Sistemas Marinos, CONICET, Bvd. Brown 2915, 9120 Puerto Madryn, Chubut, Argentina.

^3^ Universidade Estadual do Rio Grande do Sul (UERGS). Laboratório de Biodiversidade e Conservação (LABeC). Rua Machado de Assis, 1456, Osório, RS 95520-000, Brazil.

^4^ Grupo de Estudos de Mamíferos Aquáticos do Rio Grande do Sul (GEMARS). Rua Bento Gonçalves, 165, sala 1002, Torres, RS 95560-000, Brazil.

^5^ Projeto Baleia Franca, Instituto Australis de Pesquisa e Monitoramento Ambiental, Av. Atlântica, s/n - Itapiruba Norte, Imbituba, SC 88780-000 Imbituba, Brazil.

^6^ Centro Mamíferos Aquáticos, currently at Área de Proteção Ambiental (Environmental Protection Area) Anhatomirim-SC, ICMBio-MMA, Rod. SC 402, km 1, Jurerê, Florianópolis, SC, Brazil 88053-700.

^7^ Laboratório de Ecologia Espacial, Universidade do Vale do Rio dos Sinos (UNISINOS), Av. Unisinos 950, Cristo Rei, São Leopoldo, RS 93022-750, Brazil.

^8^Advanced Visualization & Geoinformatics Laboratory (VizLab), Universidade do Vale do Rio dos Sinos (UNISINOS), Av. Unisinos 950, Cristo Rei, São Leopoldo, RS 93022-750, Brazil.

^9^ Centro de Investigación y Gestión en Recursos Naturales (CIGREN), Instituto de Biología, Facultad de Ciencias, Universidad de Valparaíso, Gran Bretaña 1111, Playa Ancha, Valparaíso, Chile.

^10^ Departamento de Zoologia, Universidade Federal do Rio Grande do Sul, Avenida Bento Gonçalves, 9500, Agronomia, Porto Alegre, RS, 91509-900, Brazil

*Corresponding author: Larissa R Oliveira: larissaro@unisinos.br; orcid.org/0000-0002-5735-3697

Telefone/Phone: [+ 55 51-3591-1100](tel:%2B%2055%2051-3591-1100) - extension 1229/1278


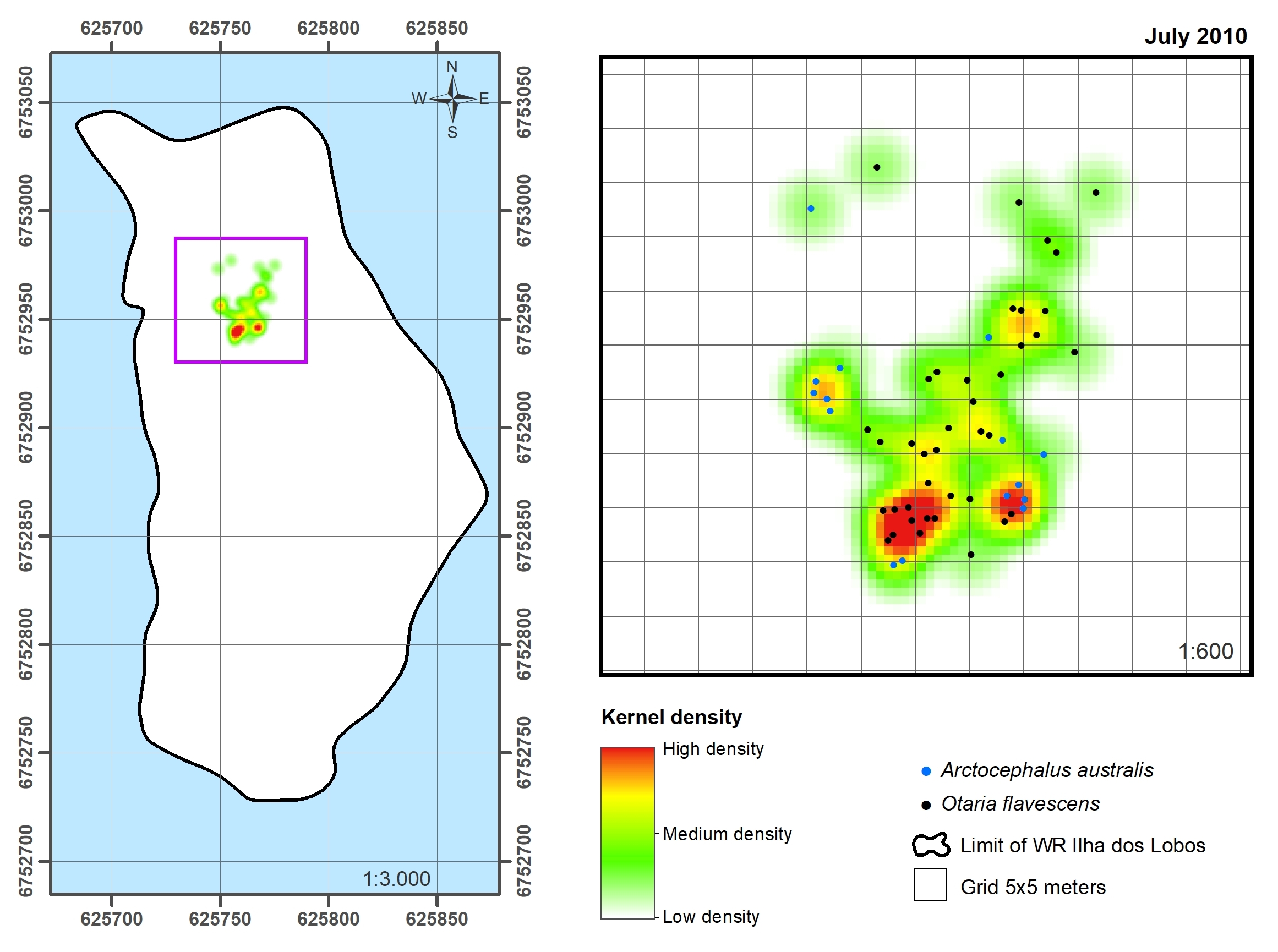


**Fig. 1.** Spatial occupation on the Wildlife Refuge of Ilha dos Lobos by pinnipeds for July 2010 with the Kernel Density Analysis (generated in ArcMap 10.6.1). Blue point: *Arctocephalus australis*; black point: *Otaria flavescens*.


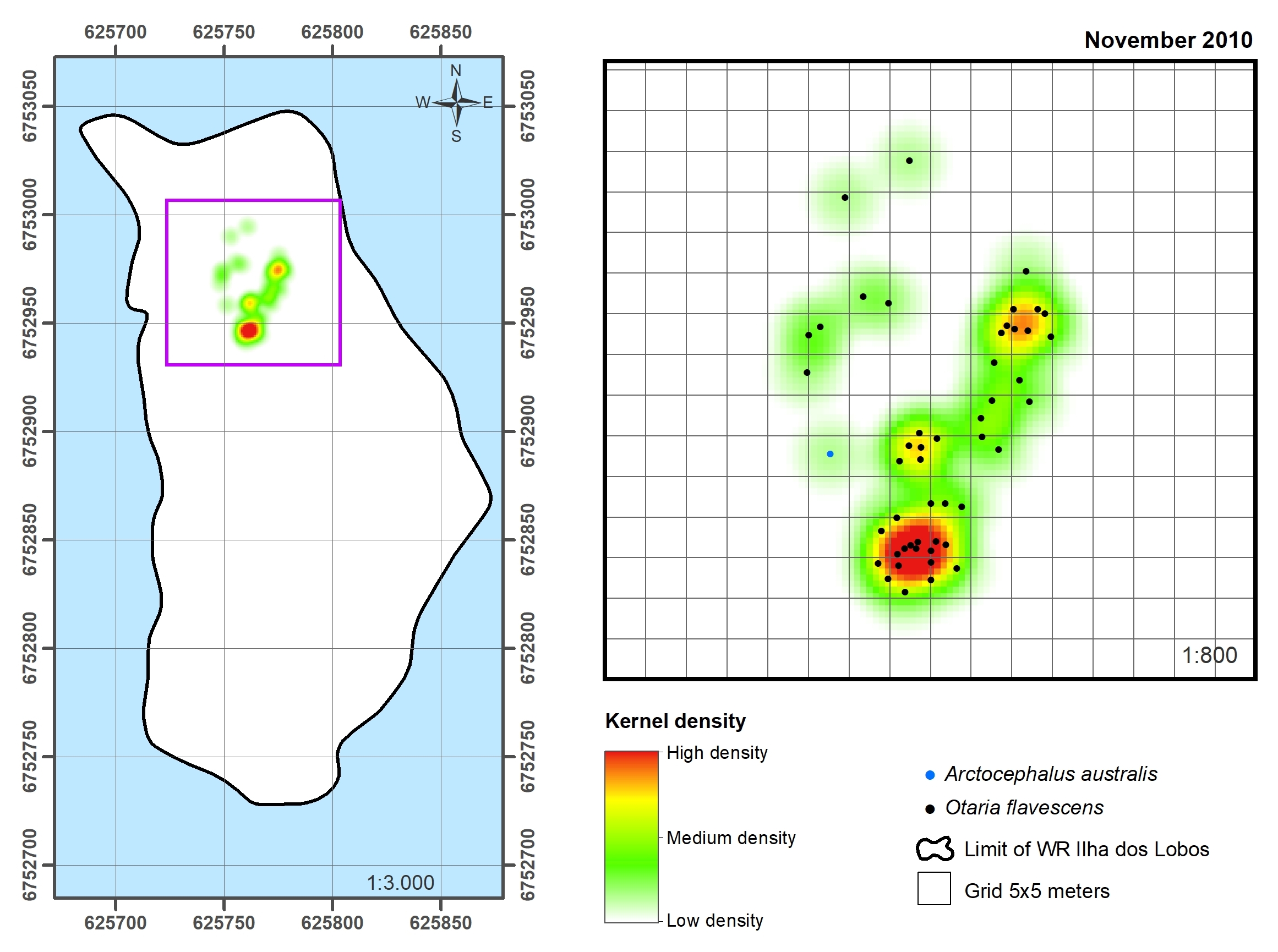


**Fig. 2.** Spatial occupation on the Wildlife Refuge of Ilha dos Lobos by pinnipeds for November 2010 with the Kernel Density Analysis (generated in ArcMap 10.6.1). Blue point: *Arctocephalus australis*; black point: *Otaria flavescens*.


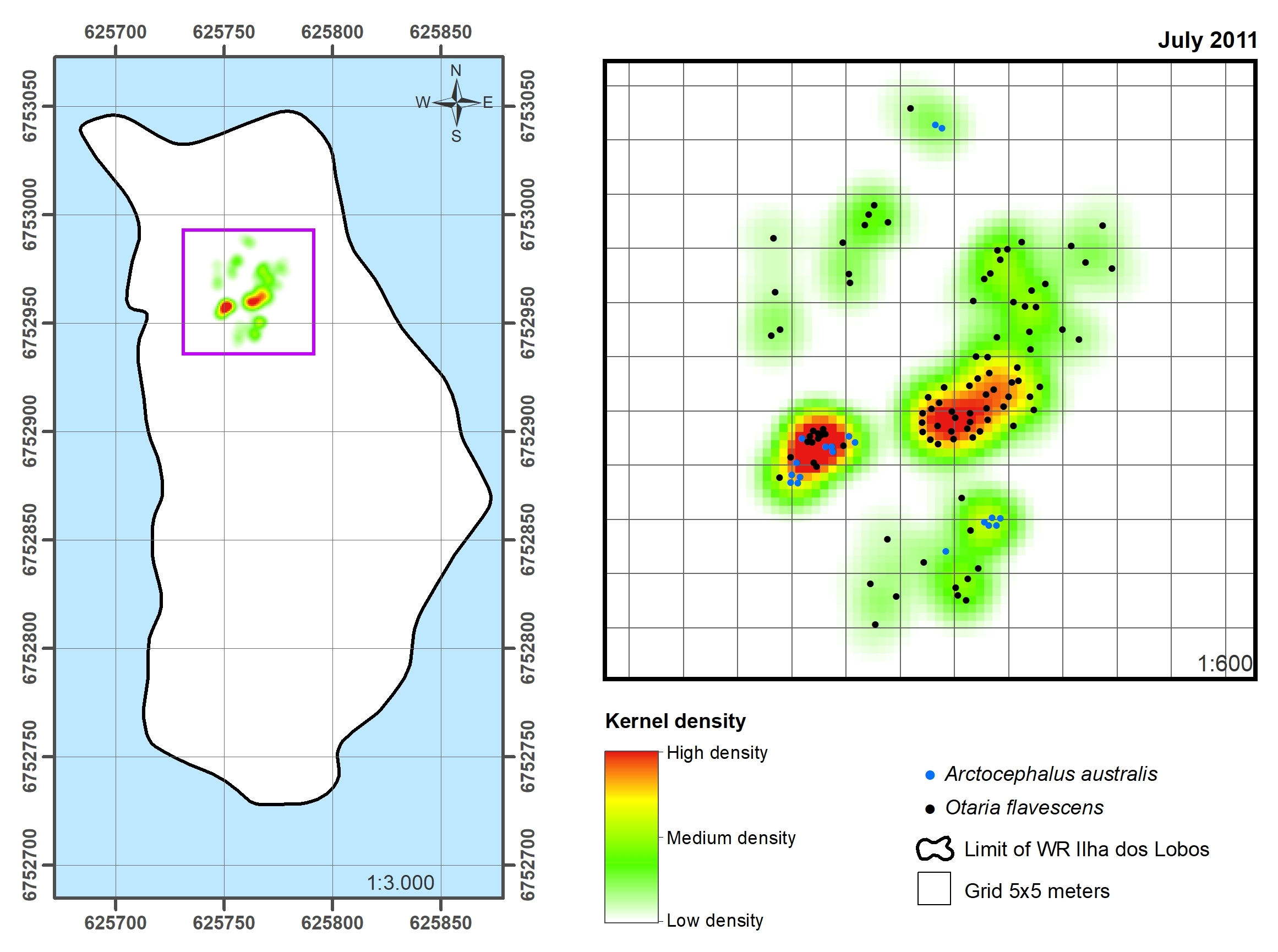


**Fig. 3.** Spatial occupation on the Wildlife Refuge of Ilha dos Lobos by pinnipeds for July 2011 with the Kernel Density Analysis (generated in ArcMap 10.6.1). Blue point: *Arctocephalus australis*; black point: *Otaria flavescens*.


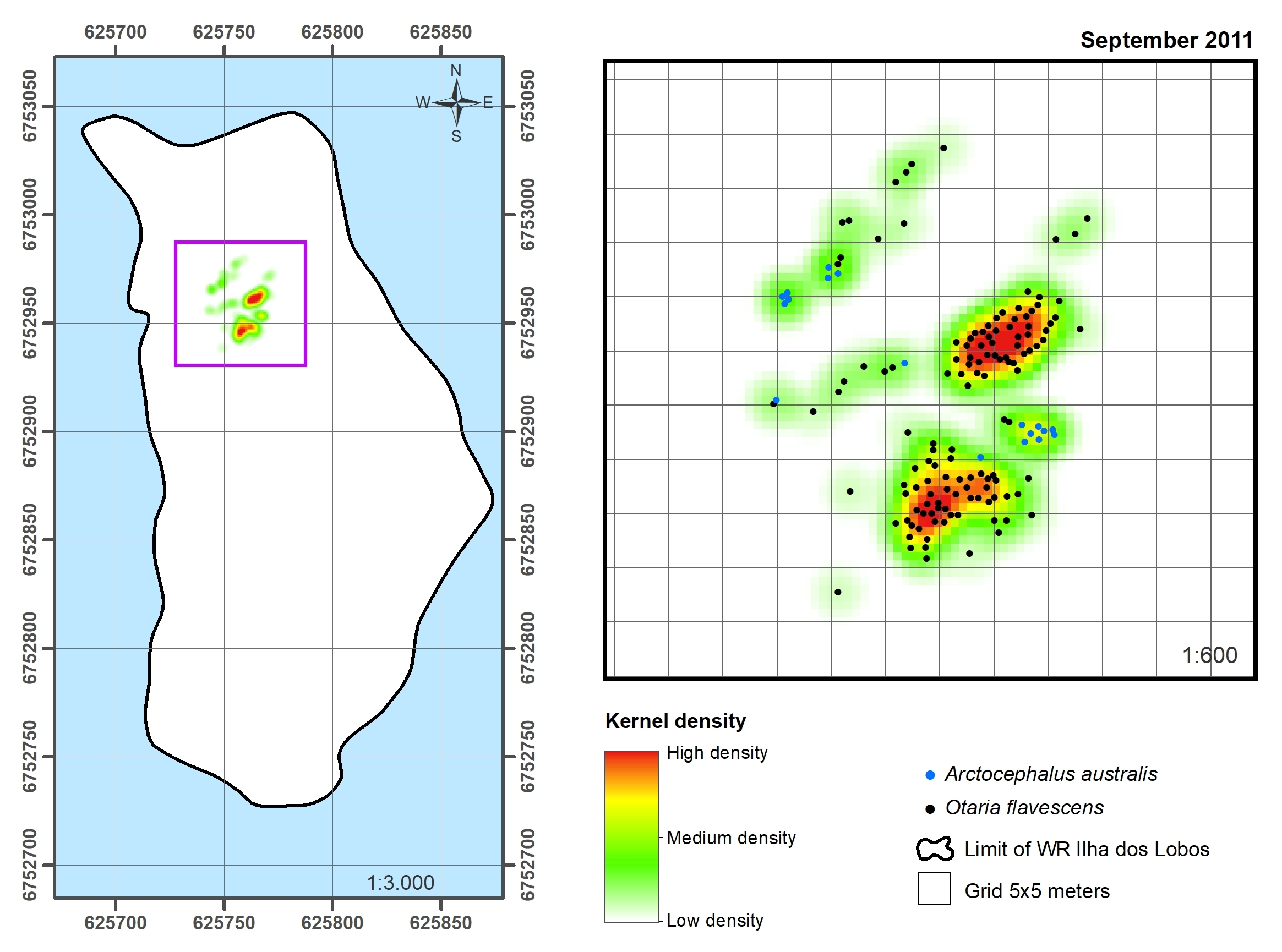


**Fig. 4.** Spatial occupation on the Wildlife Refuge of Ilha dos Lobos by pinnipeds for September 2011 with the Kernel Density Analysis (generated in ArcMap 10.6.1). Blue point: *Arctocephalus australis*; black point: *Otaria flavescens*.


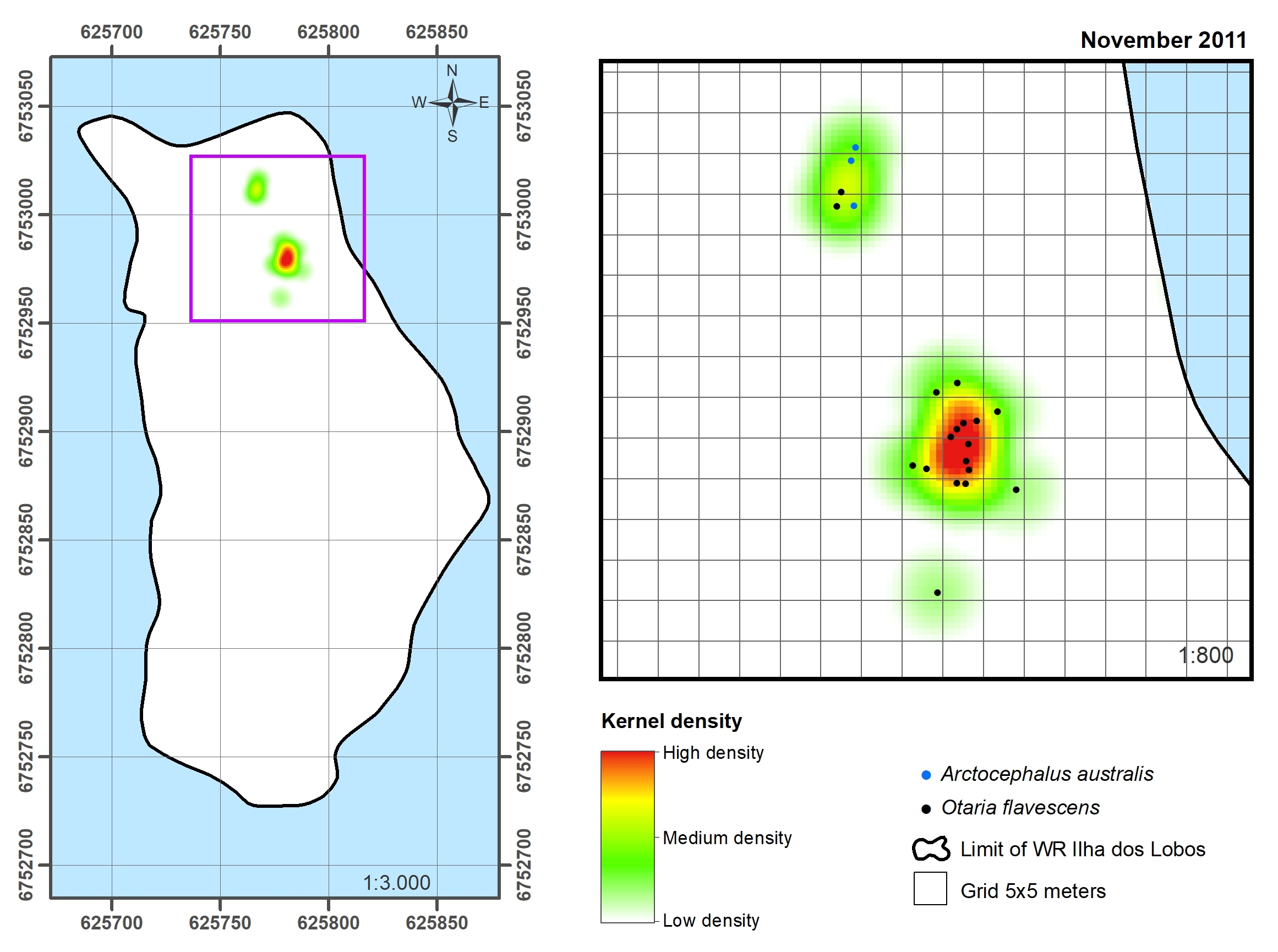


**Fig. 5.** Spatial occupation on the Wildlife Refuge of Ilha dos Lobos by pinnipeds for November 2011 with the Kernel Density Analysis (generated in ArcMap 10.6.1). Blue point: *Arctocephalus australis*; black point: *Otaria flavescens*.


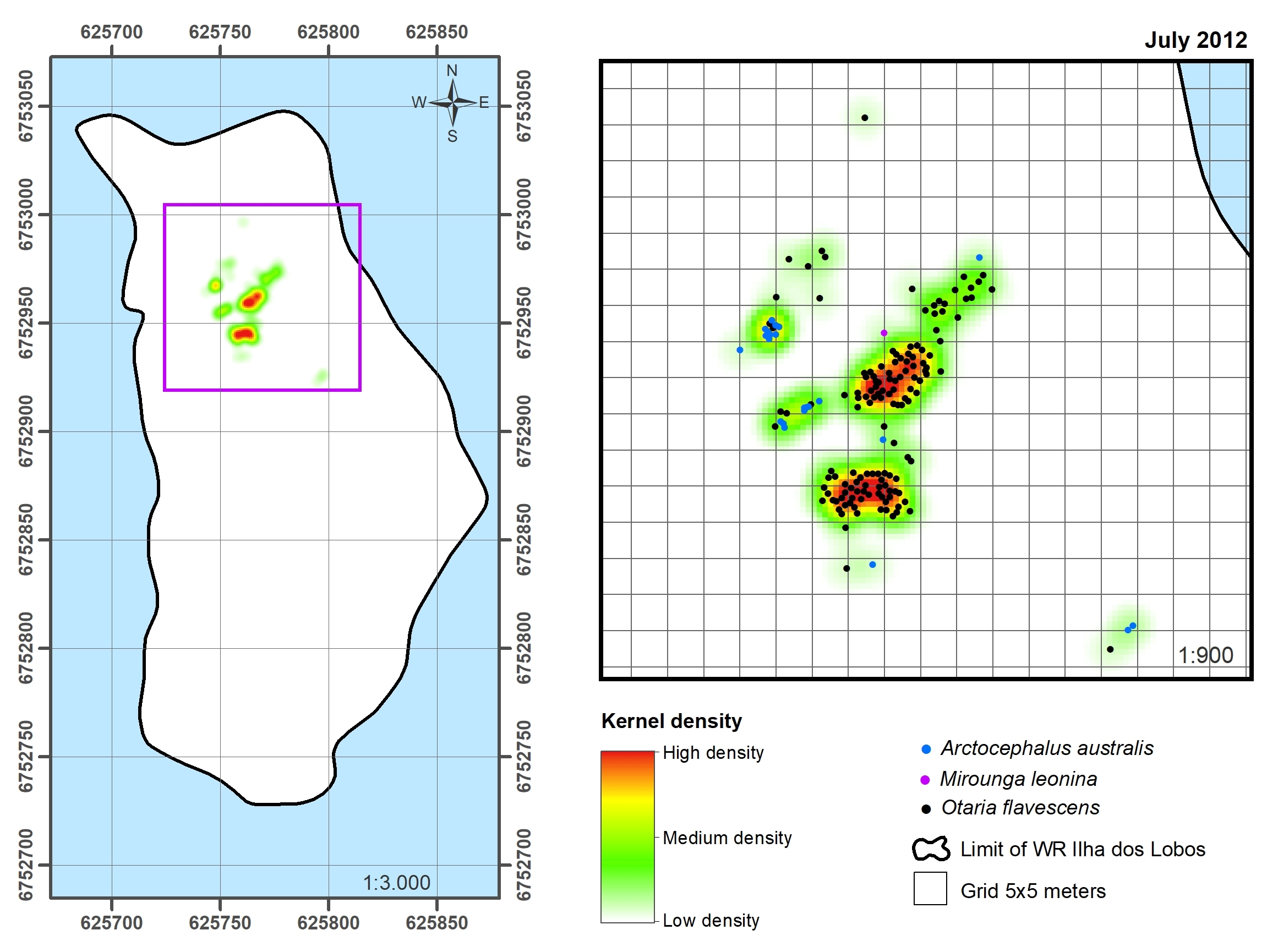


**Fig. 6.** Spatial occupation on the Wildlife Refuge of Ilha dos Lobos by pinnipeds for July 2012 with the Kernel Density Analysis (generated in ArcMap 10.6.1). Blue point: *Arctocephalus australis*; black point: *Otaria flavescens*; magenta point: *Mirounga leonina*.


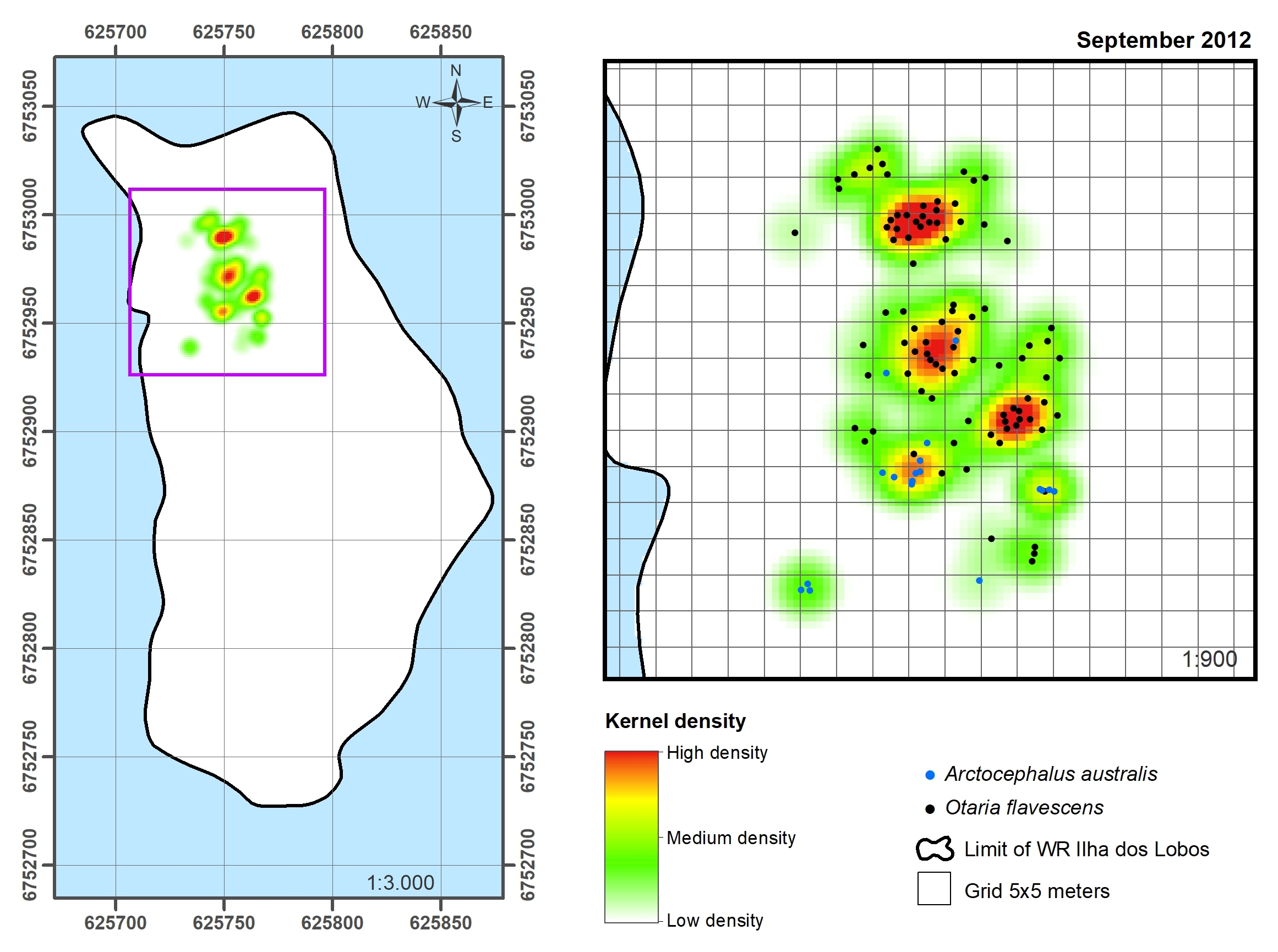


**Fig. 7.** Spatial occupation on the Wildlife Refuge of Ilha dos Lobos by pinnipeds for September 2012 with the Kernel Density Analysis (generated in ArcMap 10.6.1). Blue point: *Arctocephalus australis*; black point: *Otaria flavescens*.


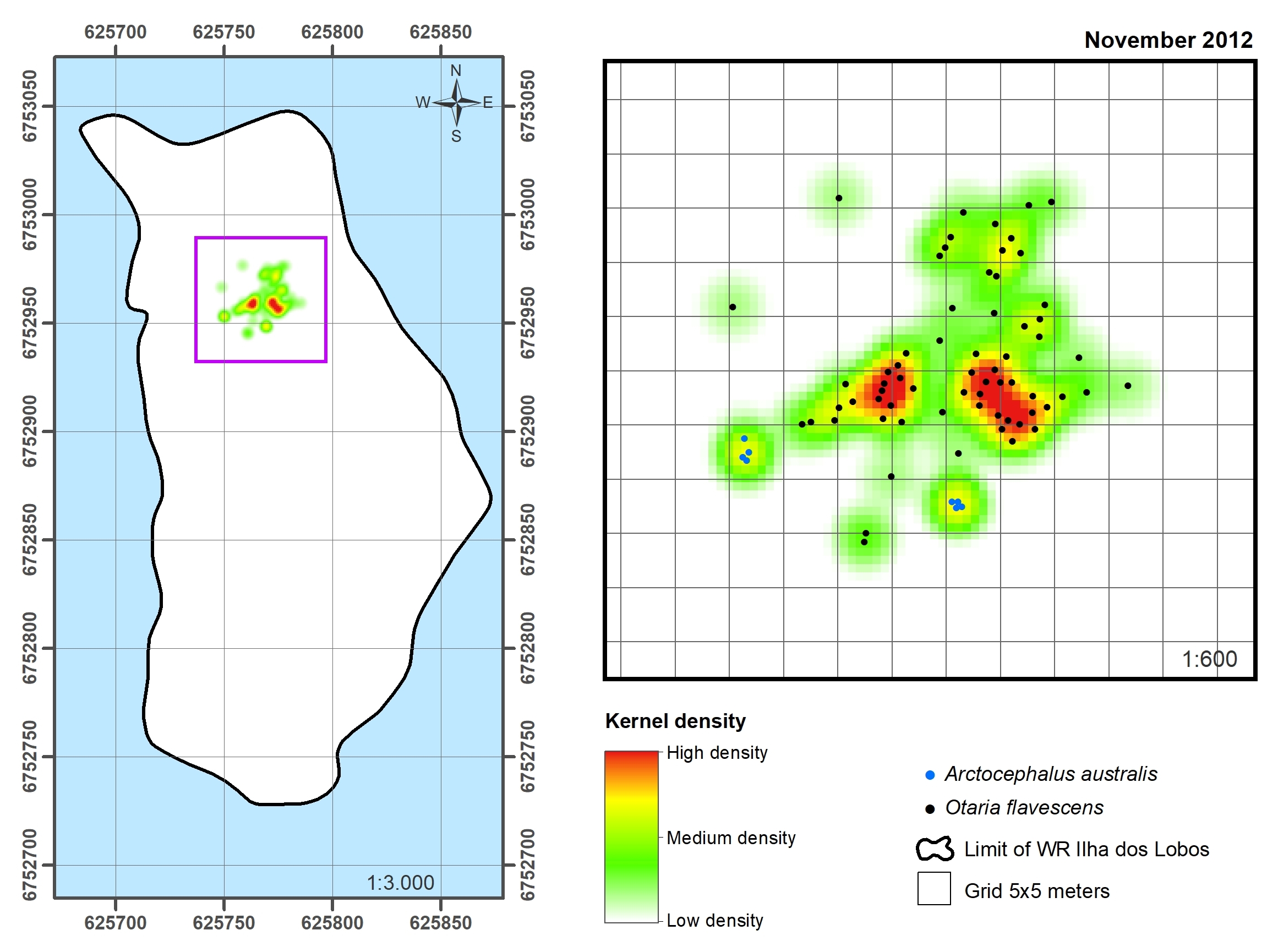


**Fig. 8.** Spatial occupation on the Wildlife Refuge of Ilha dos Lobos by pinnipeds for November 2012 with the Kernel Density Analysis (generated in ArcMap 10.6.1). Blue point: *Arctocephalus australis*; black point: *Otaria flavescens*.


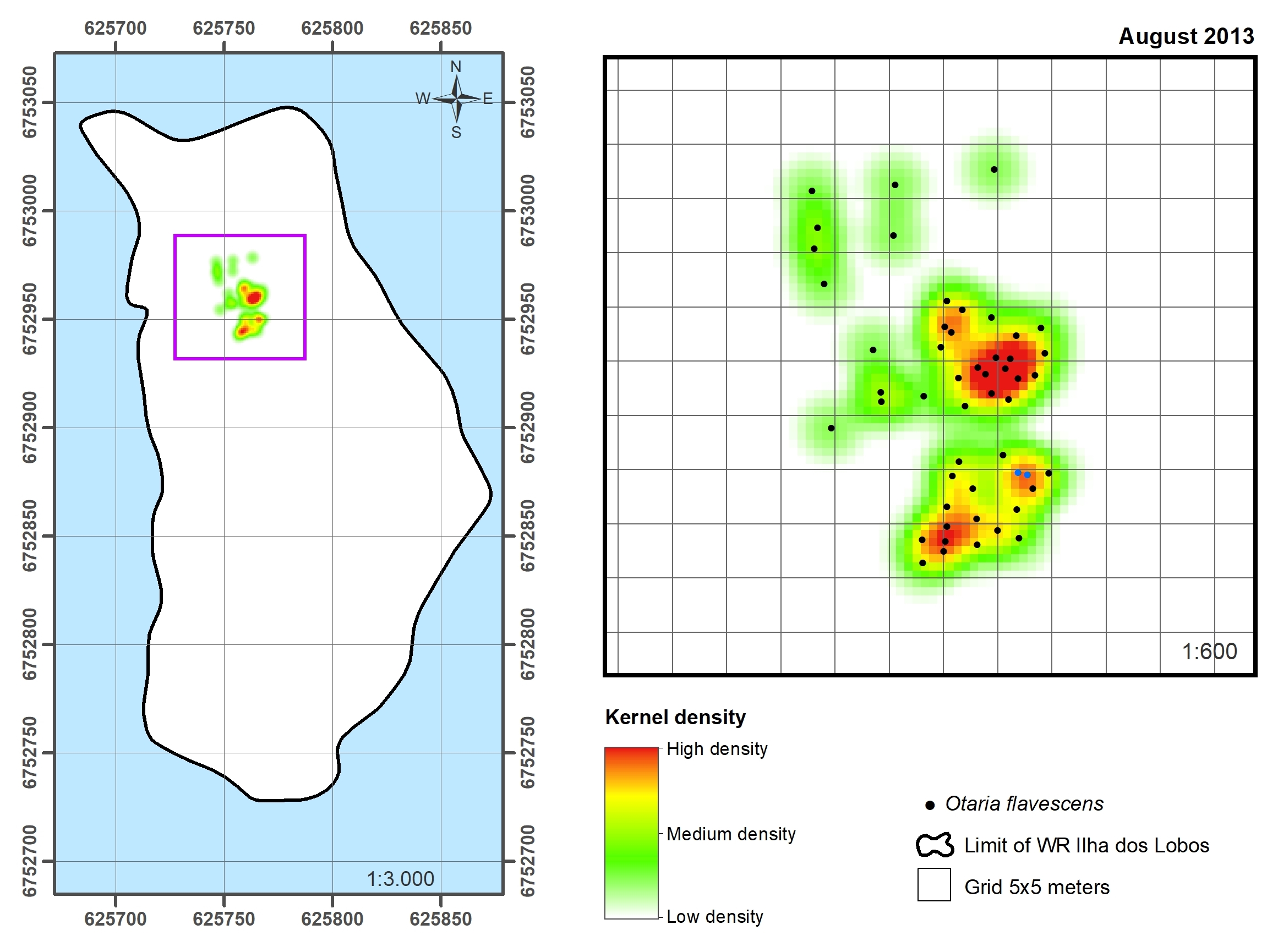


**Fig. 9.** Spatial occupation on the Wildlife Refuge of Ilha dos Lobos by pinnipeds for August 2013 with the Kernel Density Analysis (generated in ArcMap 10.6.1). Blue point: *Arctocephalus australis*; black point: *Otaria flavescens*.


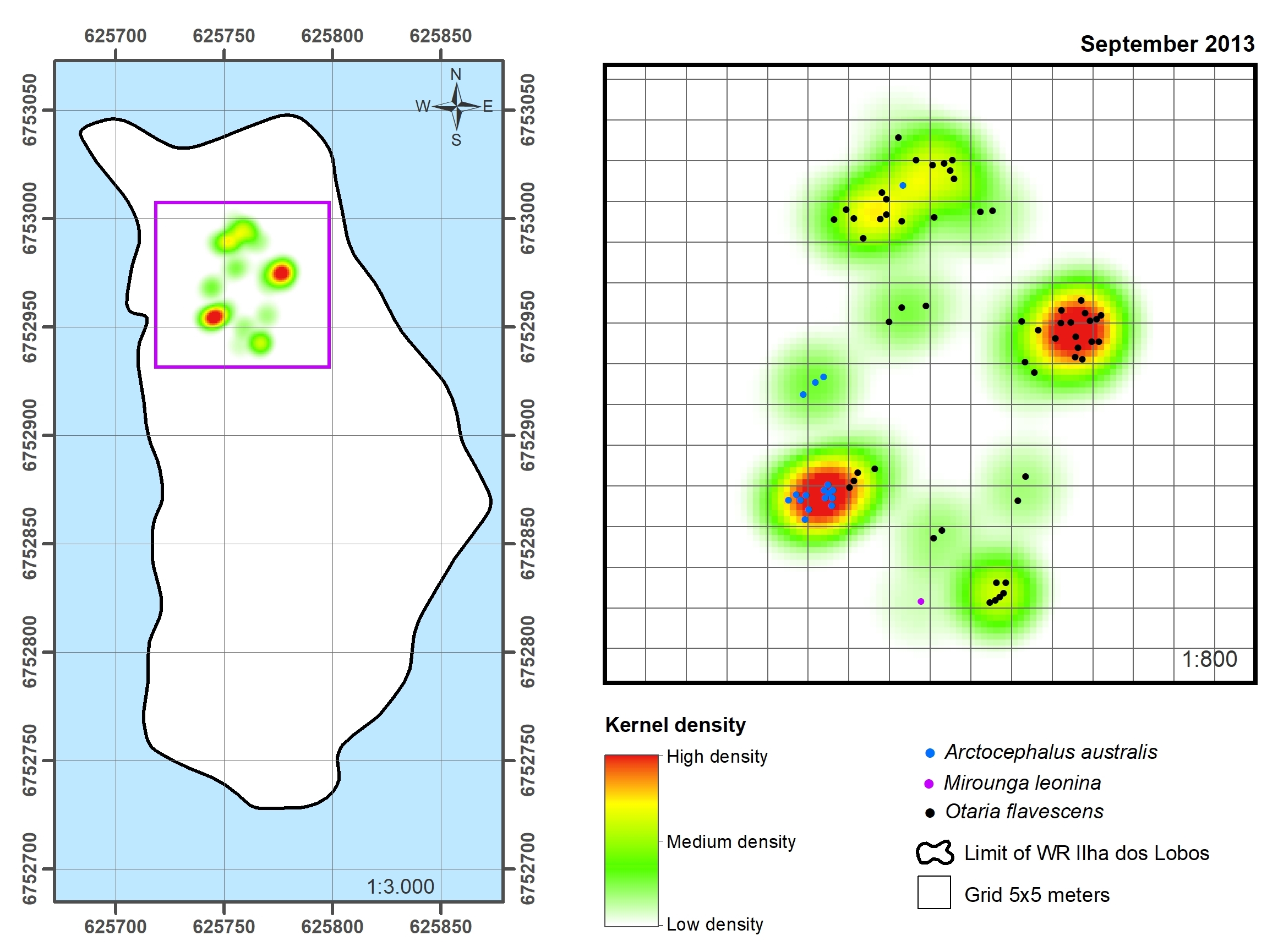


**Fig. 10.** Spatial occupation on the Wildlife Refuge of Ilha dos Lobos by pinnipeds for September 2013 with the Kernel Density Analysis (generated in ArcMap 10.6.1). Blue point: *Arctocephalus australis*; black point: *Otaria flavescens*; magenta point: *Mirounga leonina*.


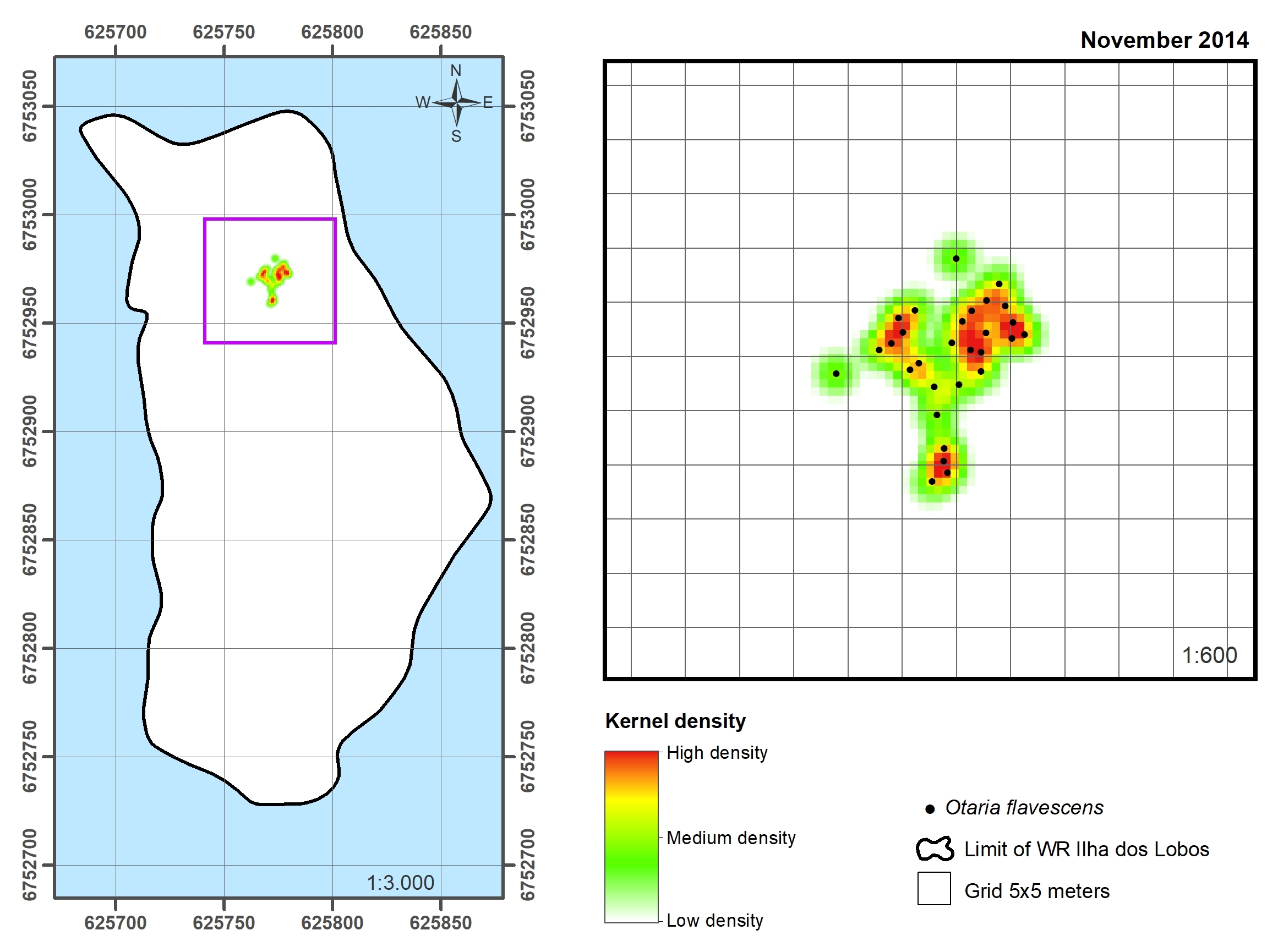


**Fig. 11.** Spatial occupation on the Wildlife Refuge of Ilha dos Lobos by pinnipeds for November 2014 with the Kernel Density Analysis (generated in ArcMap 10.6.1). Blue point: *Arctocephalus australis*; black point: *Otaria flavescens*.


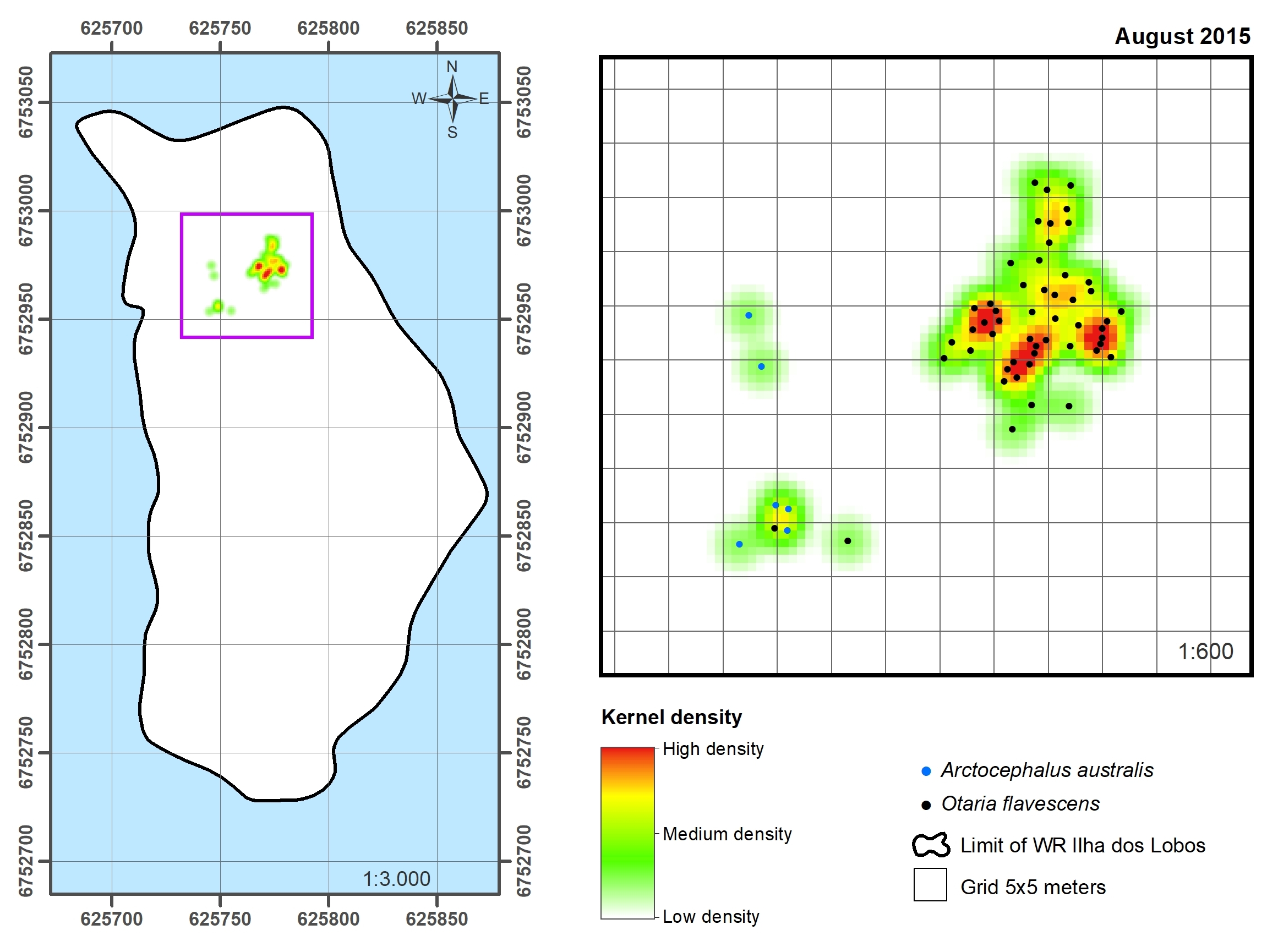


**Fig. 12.** Spatial occupation on the Wildlife Refuge of Ilha dos Lobos by pinnipeds for August 2015 with the Kernel Density Analysis (generated in ArcMap 10.6.1). Blue point: *Arctocephalus australis*; black point: *Otaria flavescens*.


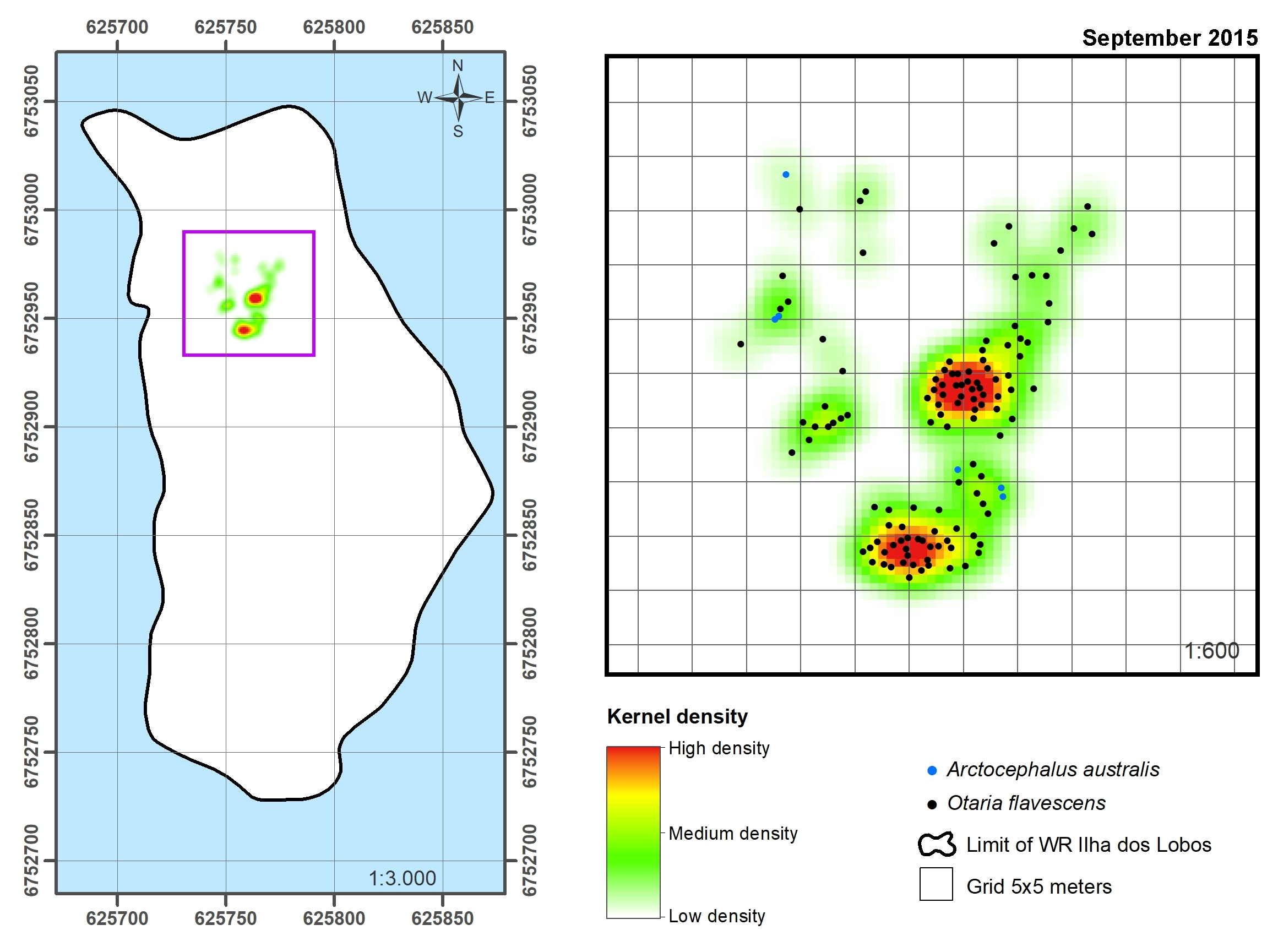


**Fig. 13.** Spatial occupation on the Wildlife Refuge of Ilha dos Lobos by pinnipeds for September 2015 with the Kernel Density Analysis (generated in ArcMap 10.6.1). Blue point: *Arctocephalus australis*; black point: *Otaria flavescens*.


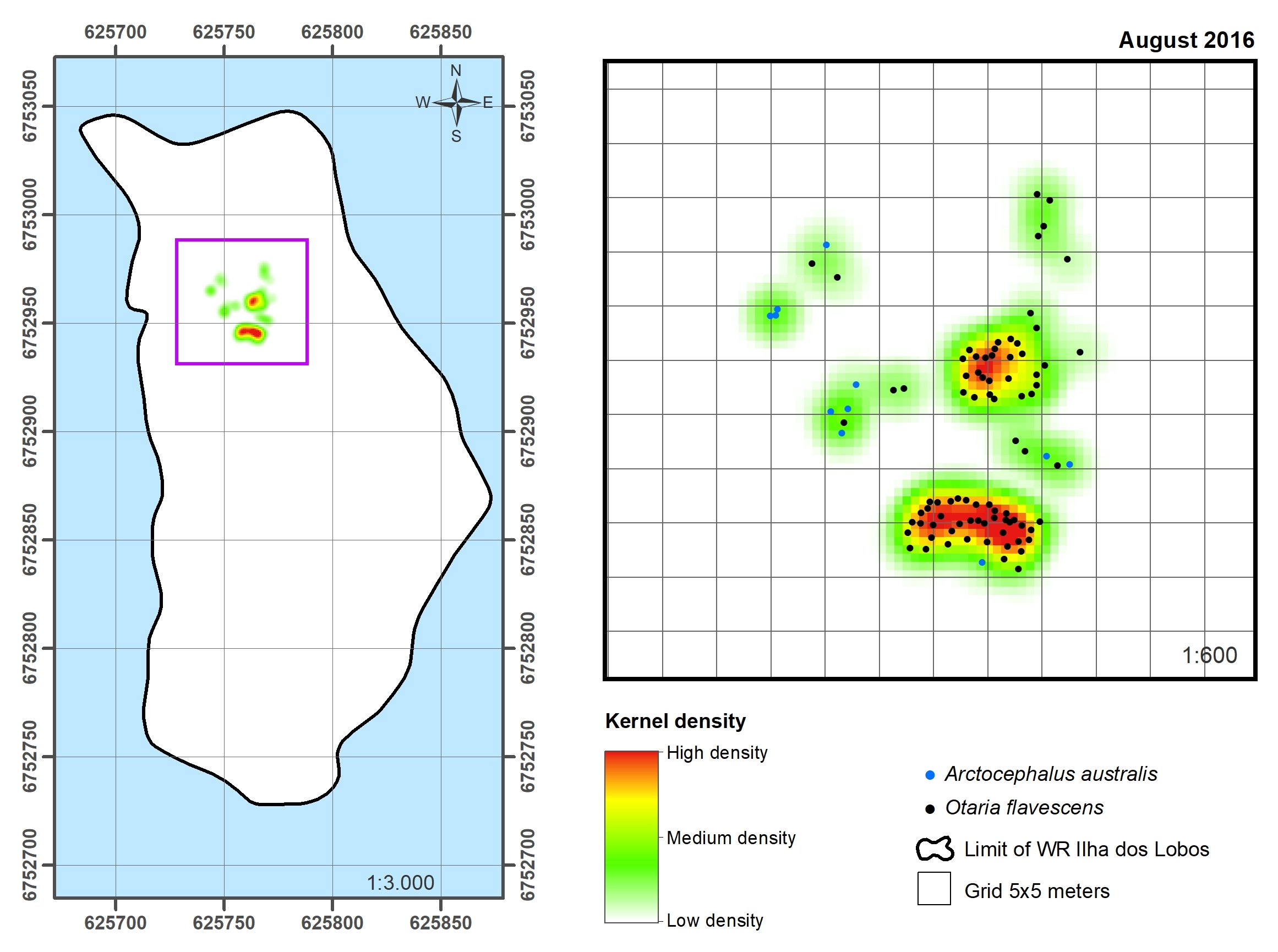


**Fig. 14.** Spatial occupation on the Wildlife Refuge of Ilha dos Lobos by pinnipeds for August 2016 with the Kernel Density Analysis (generated in ArcMap 10.6.1). Blue point: *Arctocephalus australis*; black point: *Otaria flavescens*.


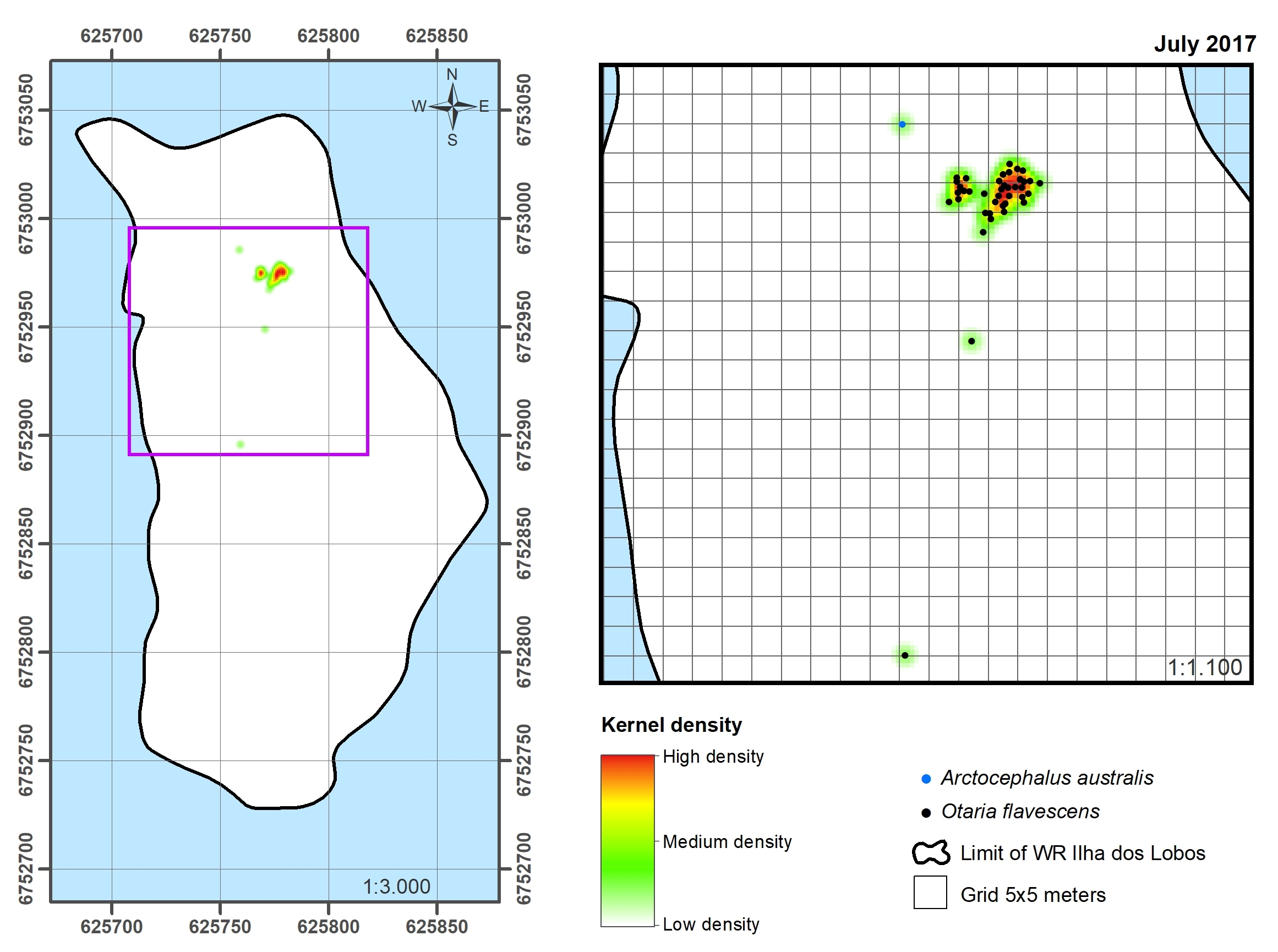


**Fig. 15.** Spatial occupation on the Wildlife Refuge of Ilha dos Lobos by pinnipeds for July 2017 with the Kernel Density Analysis (generated in ArcMap 10.6.1). Blue point: *Arctocephalus australis*; black point: *Otaria flavescens*.


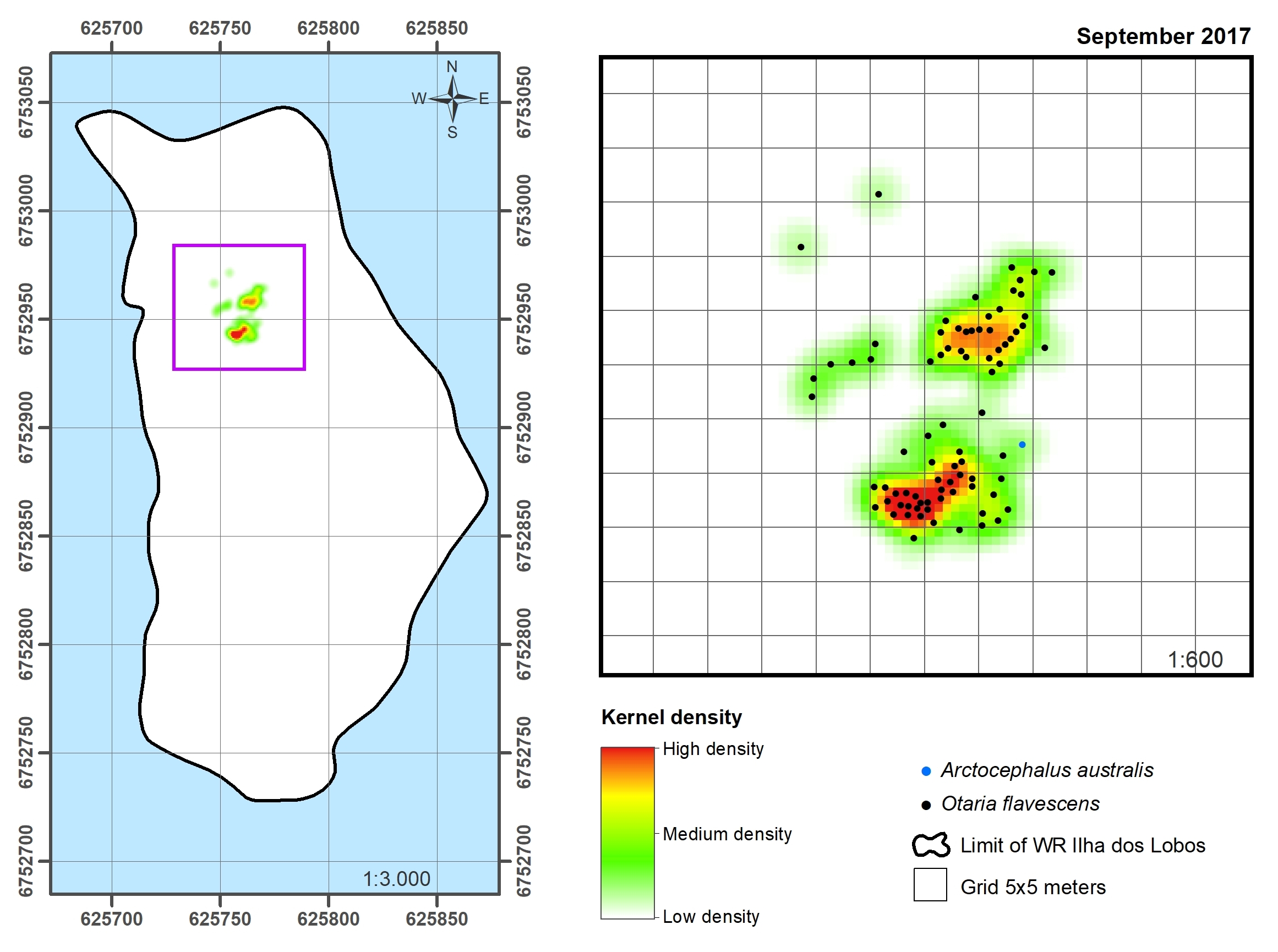


**Fig. 16.** Spatial occupation on the Wildlife Refuge of Ilha dos Lobos by pinnipeds for September 2017 with the Kernel Density Analysis (generated in ArcMap 10.6.1). Blue point: *Arctocephalus australis*; black point: *Otaria flavescens*.


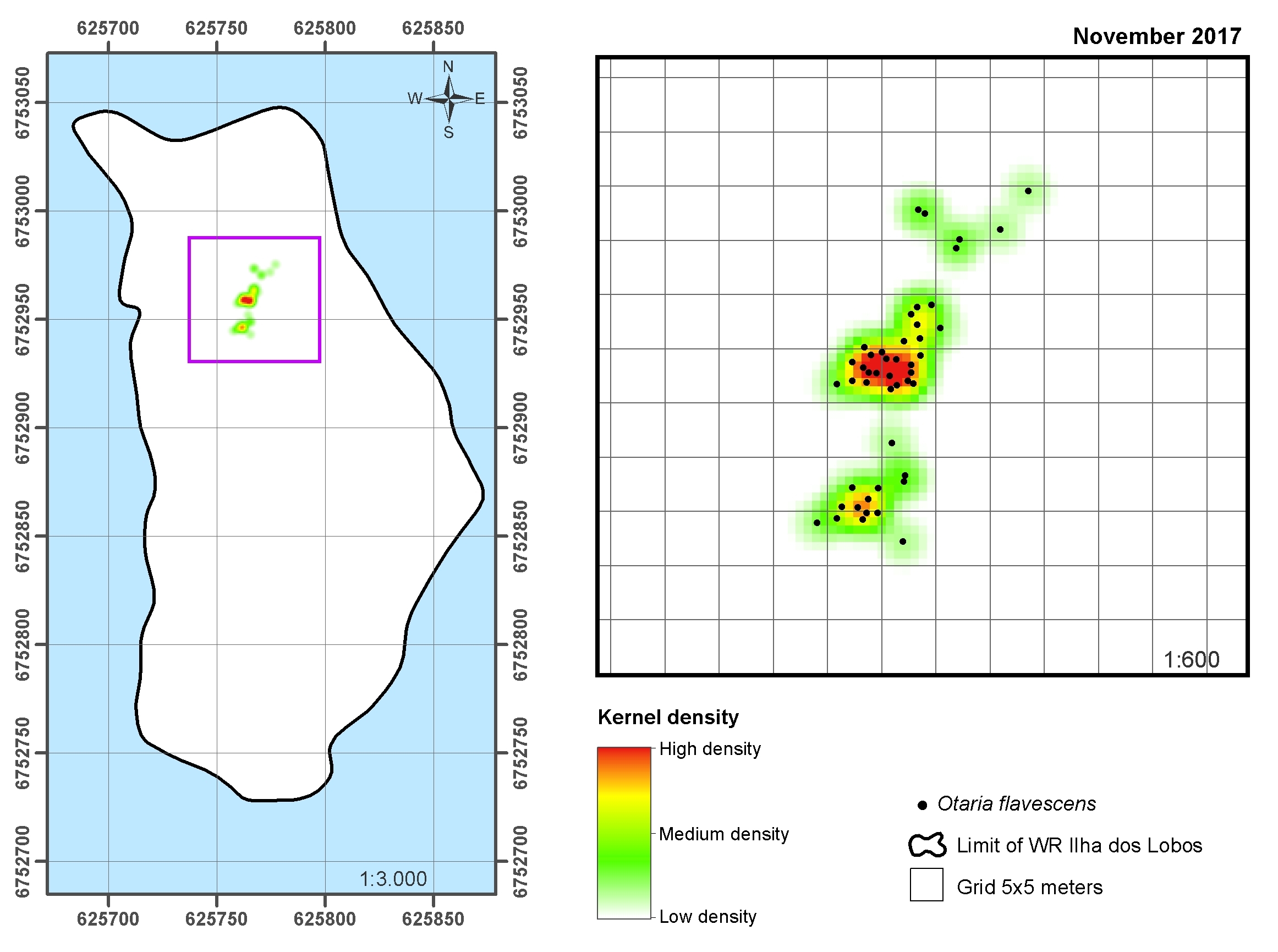


**Fig. 17.** Spatial occupation on the Wildlife Refuge of Ilha dos Lobos by pinnipeds for November 2017 with the Kernel Density Analysis (generated in ArcMap 10.6.1). Blue point: *Arctocephalus australis*; black point: *Otaria flavescens*.


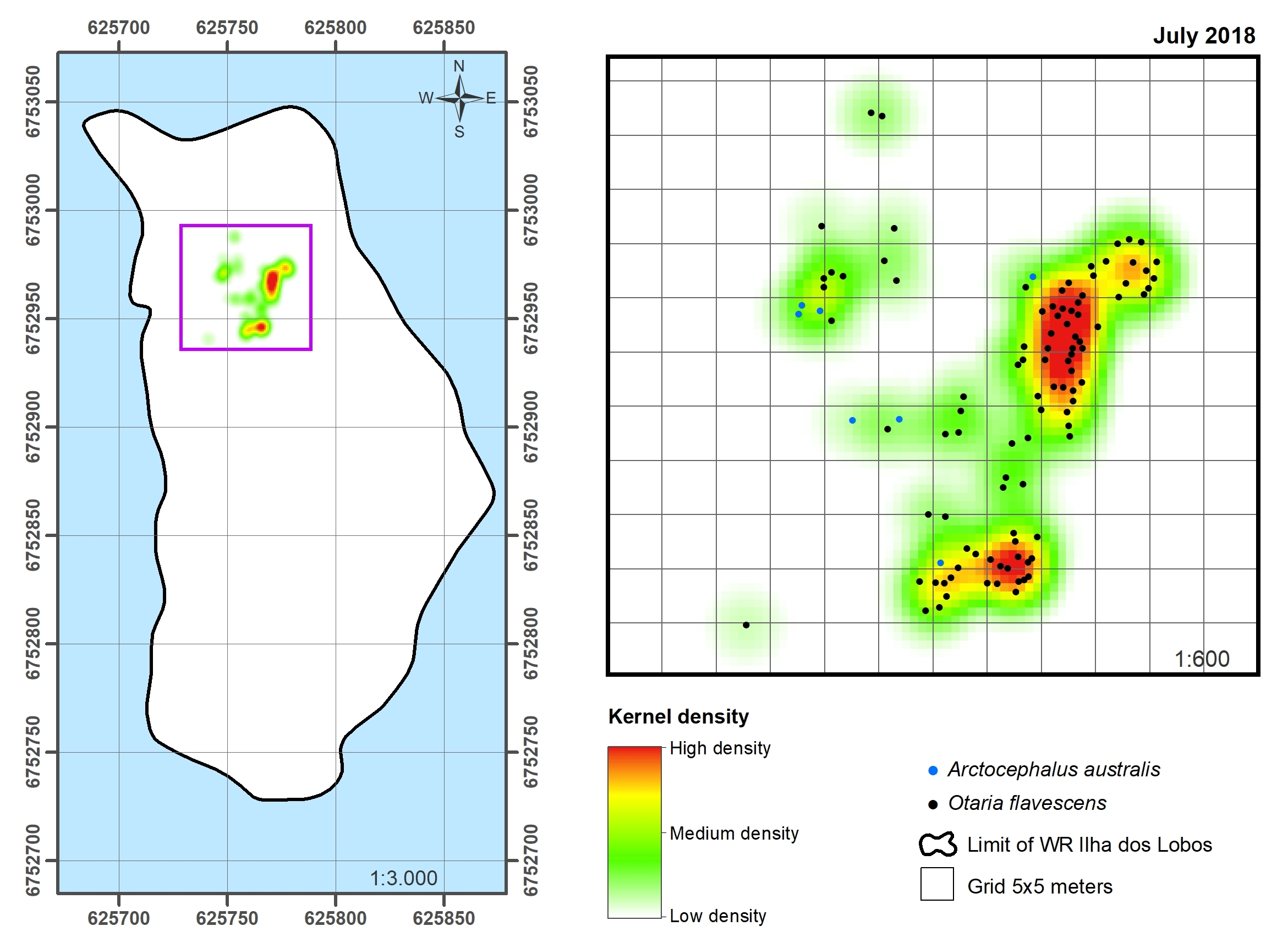


**Fig. 18.** Spatial occupation on the Wildlife Refuge of Ilha dos Lobos by pinnipeds for July 2018 with the Kernel Density Analysis (generated in ArcMap 10.6.1). Blue point: *Arctocephalus australis*; black point: *Otaria flavescens*.


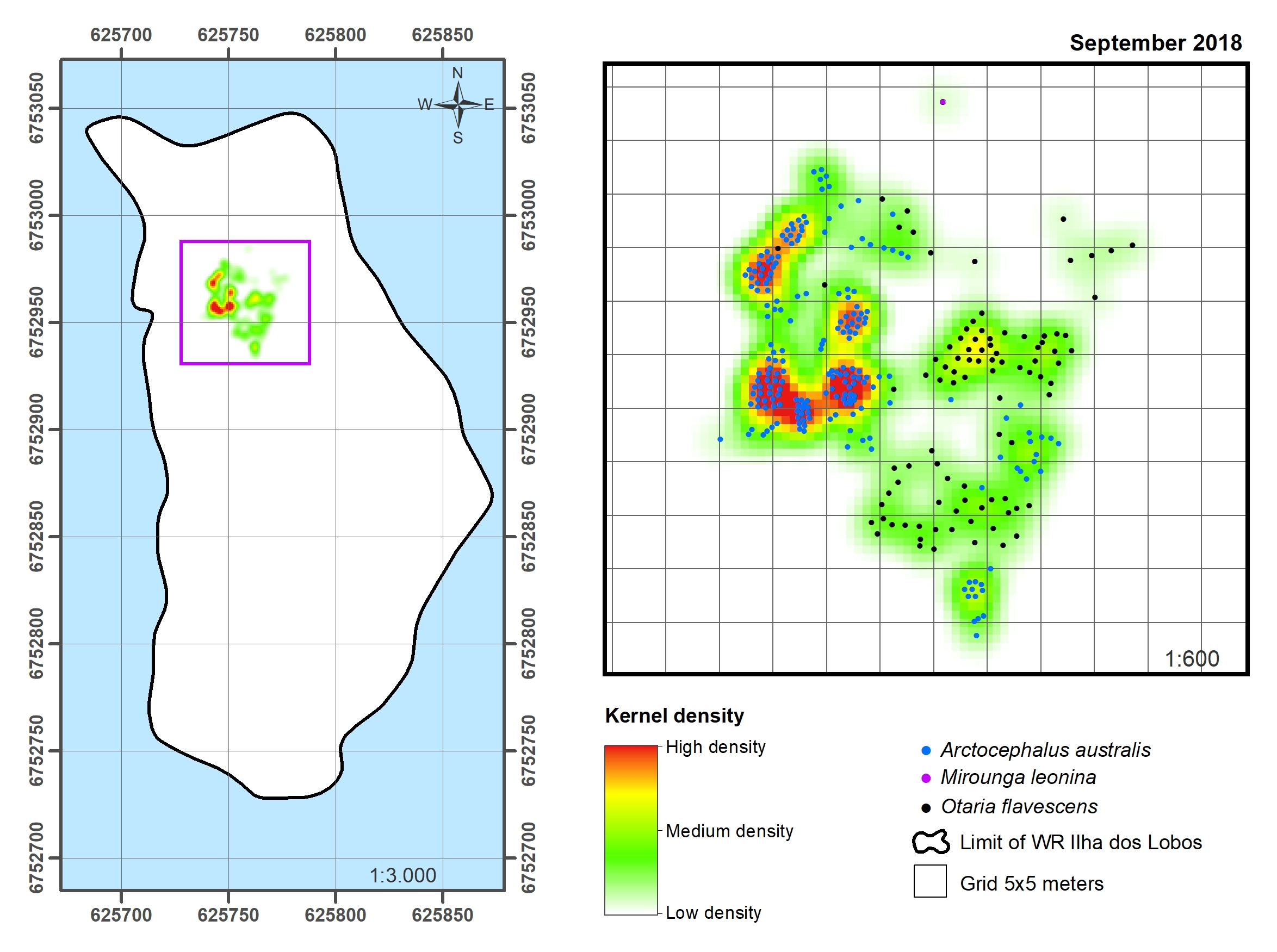


**Fig. 19.** Spatial occupation on the Wildlife Refuge of Ilha dos Lobos by pinnipeds for September 2018 with the Kernel Density Analysis (generated in ArcMap 10.6.1). Blue point: *Arctocephalus australis*; black point: *Otaria flavescens*; magenta point: *Mirounga leonina*.


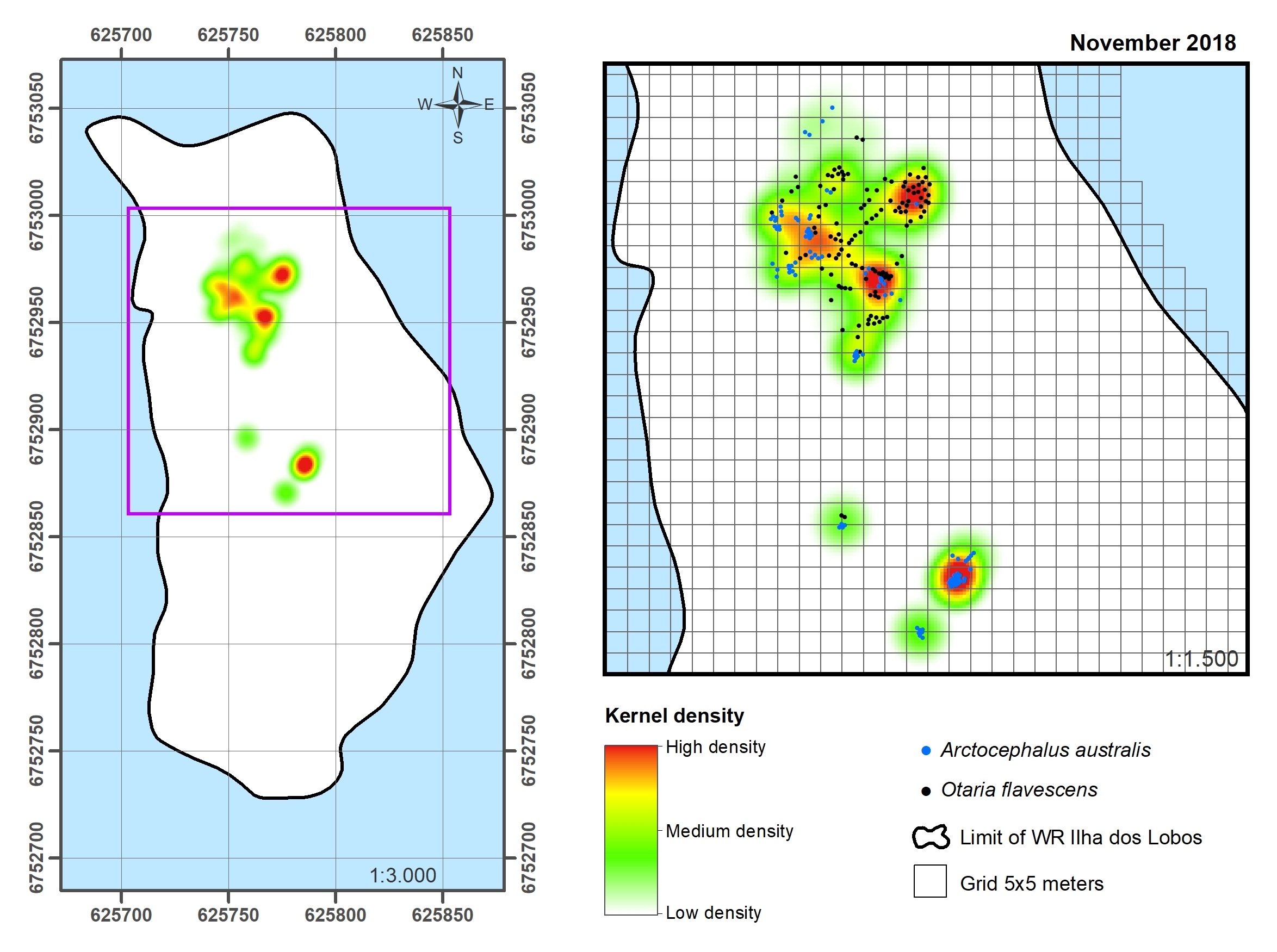


**Fig. 20.** Spatial occupation on the Wildlife Refuge of Ilha dos Lobos by pinnipeds for November 2018 with the Kernel Density Analysis (generated in ArcMap 10.6.1). Blue point: *Arctocephalus australis*; black point: *Otaria flavescens*.
